# Learnt formant modulation via upper vocal tract movements in a marine mammal

**DOI:** 10.1007/s44338-025-00145-z

**Published:** 2026-01-05

**Authors:** Teresa Raimondi, Francesca D’Orazio, Denise Di Martino, Melina Witt, Flavia Grenga, Peter Cook, Livio Favaro, Cristina Pilenga, Andrea Ravignani

**Affiliations:** 1 Department of Human Neurosciences, Sapienza University of Rome, Viale dell’Università 30, Rome, 00185 Italy; 2 Department of Life Sciences and Systems Biology, University of Turin, Via Accademia Albertina 13,, Torino, 10123 Italy; 3 Department of Marine and Terrestrial Science, University of Palermo, Via Archirafi 22, Palermo, 90123 Italy; 4 Psychology Department, Division of Social Sciences, New College of Florida, Sarasota, FL USA; 5 Master’s in Marine Mammal Science, Sarasota, FL USA; 6 Institute of Marine Sciences, University of California, Santa Cruz, CA USA; 7 Zoomarine Italia, Torvaianica-Pomezia, Rome, 00071 Italy; 8 Center for Music in the Brain, Department of Clinical Medicine, Aarhus University & The Royal Academy of Music, Aarhus, Denmark; 9 Research Center of Neuroscience ‘‘CRiN-Daniel Bovet’’, Sapienza University of Rome, Rome, Italy

## Abstract

Formants are resonance frequencies shaped by the upper vocal tract. Across vertebrates, they have a key role in acoustic communication. Humans show fine-grained control of formant modulation; little is known about such control in other species. We trained a male harbor seal (*Phoca vitulina)* to modify a baseline vocalization via upper vocal tract movements, resulting in a conditioned vocalization. The seal also had to maintain the baseline throughout the experiment. Over 150 days, we collected 455 baseline and 640 conditioned vocalizations. We extracted the first three formants (F1, F2, F3) and quantified their within-vocalization modulation using complementary measures. First, the times series of formant contours showed that F1 and F3 were similar at the start of the experiment but diverged over time. Second, coefficients of variation for F1 decreased, with higher values in the conditioned vocalizations at the end of the experiment. Third, F1 and F3 modulation depth, measuring frequency variation between adjacent time points, was higher in conditioned vocalizations. Fourth, formants’ spectral entropy decreased only in conditioned vocalizations, indicating a more predictable energy distribution over the course of the experiment. Fifth, machine learning techniques confirmed that vocal types became more distinguishable at the end than at the start of the experiment solely based on modulation parameters. Our findings show that (1) an adult harbor seal can be trained to open and close its mouth while phonating; (2) the resulting vocal output contains modulated formants and (3) differs from a baseline vocalization; (4) both vocal types’ formant features changed over time as an interconnected system.

**Supplementary Information:**

The online version contains supplementary material available at 10.1007/s44338-025-00145-z.

## Introduction

### Acoustic communication and the source-filter theory

A tenet of vertebrate bioacoustics is the source-filter theory of vocal production. This framework conceptualizes sound production as a three-stage process. Air is expelled by lungs. The vocal folds (sound source) vibrate due to these puffs of air and produce a fundamental frequency with its harmonics. The upper vocal tract (filter) acts as a sort of sound equalizer; its morphology dampens these frequencies to different extents. This way, the upper vocal tract produces a set of *formants.* A single formant is defined as a “vocal tract resonance and/or a prominent spectral peak resulting from such a resonance. The relative positions of the first three formant frequencies (F1 to F3) determine the vowel quality in speech, whereas formant spacing among all measurable formants can be used to estimate apparent vocal tract length” [1, p.2].

### Features and functions of formants

Formants have an enormous biological and psychological importance [1–7]. Across species, formants can encode body size [8], aggressive intents [9], individual identity [10], and many more features of a vocalizing animal [1, 11]. Some of these are invisible to the eye of the receiver of the signal, suggesting that in some cases acoustic formant production and perception are the only means of communicating a specific piece of information [12, 13]. In human behaviour, formants are also crucial. In spoken language, formants make the difference between any two vowels [14]. In adult laughter formants shift upwards to high frequencies [15], and in infant cry they undergo a developmental tuning [16]. Finally, music provides an example of adult tuning of formants: singers, in fact, learn to arrange their vocal tract in order to enhance specific harmonics produced by their larynx [16, 17].

Across species, including humans, the three formants with lowest frequency - F1, F2 and F3 - are the most important ones, both methodologically and biologically. Methodologically, under the reasonable assumption that the source’s harmonics have decreasing energy as their frequency decreases, F1, F2 and F3 are the easiest to detect on a spectrogram [18]. For partly overlapping reasons, biologically, lower formants are also the most straightforward to produce and perceive. Theoretically, for most vertebrates, processing the first two formants may be enough to make an educated guess on the sex [19], arousal [20] or intentions [9] of the emitter. In human spoken language, F1, F2 and F3 encode most of the information necessary to discriminate among vowels [17].

### Vocal tract movements, formant modulation and neural control

F1, F2, and F3 can encode information, as has been documented in numerous mammalian species. For instance, the formants of a koala’s bellow are an honest signal of their body size [21]. Similarly, deer use formants in other deer’ vocalizations to infer the vocalizer’s body size and behave accordingly [9]. *Dynamic formant filtering* is however less common across species. Here we define it as the capacity to dynamically change the configuration of the vocal tract to alter formants, within a single vocal type or vocalization context. Only in 2001, the first non-human case of descended larynx was discovered: red deer lower their larynx from the resting position while vocalizing [22]. This example however constitutes a one-off movement which barely makes use of the communicative potential of the formant space. In other cases, such as dogs barking, one may hypothesize that mouth opening and bark emissions are coupled; based on current evidence (e.g [23]), it may be hard for a dog to bark while closing, instead of opening, their mouth. Are there examples of dynamic formant filtering, where animals control their dynamic tract configuration to modulate sounds?

At least in human vocal communication, the species for which we know most about formants, most information is conveyed by dynamic formant filtering. Our species *learns to modulate formants* by learning motor sequences for the upper vocal tract [24]. This in the past led to the question: is an adequate vocal tract morphology enough for a mammal to modulate its formants? Work on macaques suggested that vocal tract morphology only provides a weak constraint on formant production and modulation; in other words, several vocal tract morphologies are allowed but the crucial requirement for the species in question is to have the right neural wiring to allow for the modulation [25]. In addition, any given species may have the right neural connections for formant modulation but simply not show this behaviour spontaneously or frequently enough to be detected. Enter training via operant conditioning: Several species can be asked to modify their vocal features by shaping a spontaneous vocalization through rewards [26–28]. Crucially, however, not everything can be trained, and these results bear relevance to the species’ biology beyond the lab: as one cannot train dogs, who are color-blind, to discriminate between green and red, one cannot show trained formant modulation in a species lacking the adequate neurobiological underpinnings.

### Phocid pinnipeds and previous work

Among the >6600 species of mammals, one group seems particularly promising to find abilities to (learn to) modulate their formants dynamically [29–33]. The phocids are a family of aquatic mammals belonging to the order of carnivores, parvorder pinnipeds. Behavioral evidence on a phocid species suggests that the harbor seal (*Phoca vitulina*) can act on each of the 3 components of the source-filter framework when learning sounds [34–43]. Neurobiological evidence suggests that, among pinnipeds, they may have the most apt white-matter wiring necessary for this endeavour [29]. For the particular case of *learning how to modulate formant properties* of emitted sounds, two main pieces of evidence exist to date. Hoover, a harbor seal, had learnt to imitate human speech; however, while Hoover’s spectrograms show formant modulation, no quantitative formant measurements were analyzed [34, 42]. This makes Hoover’s case a promising anecdotal piece of evidence rather than a solid dataset on which to base inference. A second case saw the harbor seal Sprouts trained to open and close its jaw while producing sounds [28, 40]. This was one of the first studies of its kind in a non-human mammal, showing that the degree of jaw opening correlates with the frequency of F1 in the resulting sound. The study however had a few limitations: the training process was not documented longitudinally; there was no other baseline vocalization to compare the trained vocalization with; only the first two formants were extracted and analyzed.

### Our goals, questions and hypotheses

Based on the above, we set out to answer some questions: Does the dynamic formant modulation seen in Sprouts replicate in another individual harbor seal? How does the learning process develop longitudinally and compared to other vocalizations in the individual’s repertoire? Can other acoustic parameters, beyond the bare formant frequency, capture nuances in the process?

Specifically, we had 3 main goals. First, we wanted to show that harbor seals *can*, under the effect of operant conditioning, learn to volitionally control their formant modulation. Second, we wanted to replicate previous results showing *dynamic modulation* in one harbor seal, with variations on the richness of extracted acoustic measures and the addition of a baseline vocalization. Third, we wanted to track and describe this process longitudinally.

**Fig. 1:**
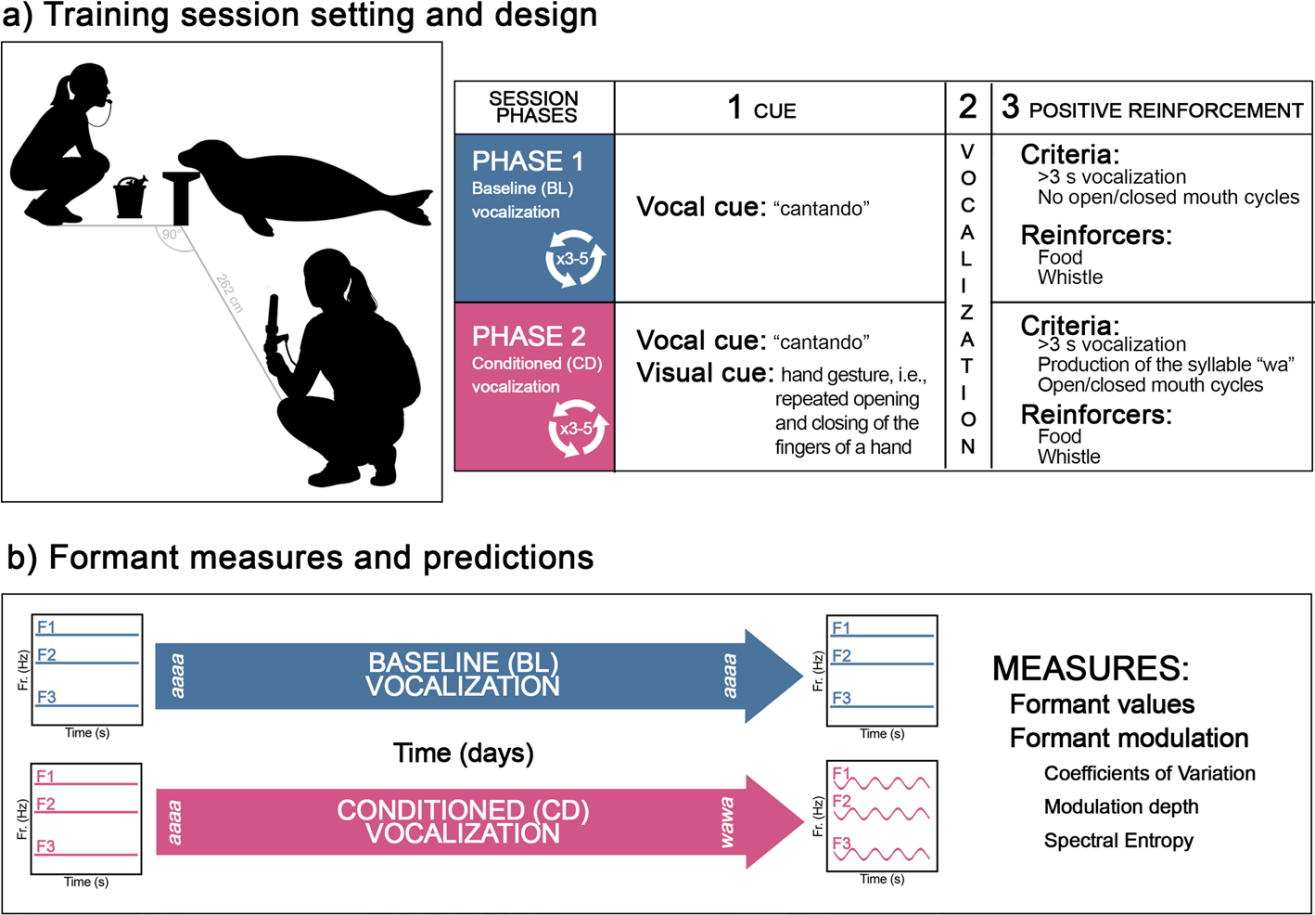
**a** Training session setting and design: Every session consisted in two phases. In the first phase, baseline (BL) vocalizations were elicited using the vocal cue *“cantando”* and reinforced if they lasted longer than 3 s without mouth cycles. In the second one, conditioned (CD) vocalizations were elicited using the vocal cue *“cantando”* together with a visual hand gesture, and reinforced if they lasted longer than 3 s, included open/closed mouth cycles, and produced the repetition of the syllable “wa”. Reinforcers included food and whistle signals. **b** Used formant measures and predicted outcome: Acoustic analyses were conducted on BL and CD vocalizations across time, focusing on formant values (F1, F2, F3) and formant modulation (coefficients of variation, modulation depth, and spectral entropy).As schematized in the spectrograms, at the beginning of the experiment, both vocalizations are expected to be similarly non-modulated in formant modulation measures. At the end of the experiment, modulation of formants is expected to increase in CD but not BL vocalizations

To achieve these goals, we worked with a seal previously trained to phonate on command, producing what we call the *baseline vocalization* (BL; Fig. 1a). Prior to this study, no selective shaping of this vocal response had been operated, but the cued output was fairly stereotyped. Starting from BL, we then trained the seal to produce a second *conditioned vocalization* (CD). Unlike the BL vocal type, the CD was trained as phonating while opening and closing the mouth. From the audio recordings, for each formant we extracted its *formant contour*: the time series of frequency values corresponding to a specific formant. Based on these formant contours, we performed 5 types of analyses to address the questions above. For each recorded BL and CD vocalization, the 5 analyses involved (Fig. 1b):

1. Values of the first three formants. The extracted formant values capture the overall spectral placement of each formant. They were sampled at constant intervals and linked to form continuous contours representing the temporal evolution of F1, F2, and F3 separately.
2. Coefficients of variation (CV) of formant values, reflecting frequency stability within a vocalization: the higher the variation of each formant contour, the higher their modulation.
3. Modulation Depth, measuring the absolute difference in frequency between adjacent points along each formant contour, quantifies frequency jumps per time unit; it reflects how steep or rapid the contour changes are. Higher values indicate sharper modulations.
4. Spectral Entropy, indicating the predictability of spectral energy distribution: the more predictable the formant contour modulation, the lower the entropy. Cyclic repetition of vowel-like sounds corresponds to higher predictability of the formant contour compared to a random trajectory of formants.
5. Dimensional reduction on the modulation measures (2–4) and vocal types’ classification to quantify their separation in the acoustic space, at the start vs. at the end of the experiment.

## Materials & methods

### Study site

Data was collected at Zoomarine (Torvaianica, Italy), a zoological facility covering approximately 40 hectares, which is home to nearly 380 animals from 37 different species. Zoomarine is officially recognized as a zoological garden under Italian National Law 73/2005. The seals and other animals are trained through operant conditioning using positive reinforcement, encouraging voluntary cooperation during medical and veterinary procedures [44]. The pinniped facility comprises 11 water tanks, each partitioned into a water zone and a dry zone, designed to meet the particular needs of the animals. The tanks are all filled with artificially salted water with food-grade washed sea salt; filtration is closed-loop with a mechanical filter, sterilization is by medium-pressure UV lamps, and chlorine addition is < 0.8 ppm free.

### Subject and baseline vocal behaviour

The subject of this study, named Tattoo, was a seven years old, adult male harbor seal (*Phoca vitulina*). It was born in managed care at the “Le Cornelle” wildlife park in Italy (Valbrembo, Bergamo), on June 8, 2017. A few months after its birth, the transfer of the captive-born specimen has been done on a donation agreement basis provided by the European program of exchange of animals in the accredited zoos. The subject currently resides in Zoomarine in a mixed group of pinnipeds, including harbor seals, grey seals, and sea lions. The animals are rotated daily among pools and social groups, promoting variable social dynamics and consistent environmental enrichment. Throughout its life, the subject would spontaneously produce vocalizations that, throughout the years, the trainers placed under the control of the discriminative vocal cue “cantando”, positively reinforcing its emission in specific contexts related to the park’s public entertainment and dissemination. Without shaping its acoustic features, over time, the trainers selectively reinforced the seal to produce only this vocalization type, which has been part of his training repertoire since that time. Crucially, this vocalization did not match, in context of emission and acoustic properties, any of the known vocal types of the repertoire of an adult harbor seal in the wild or ex-situ [45, 46].

### Operant conditioning procedure

**Fig. 2:**
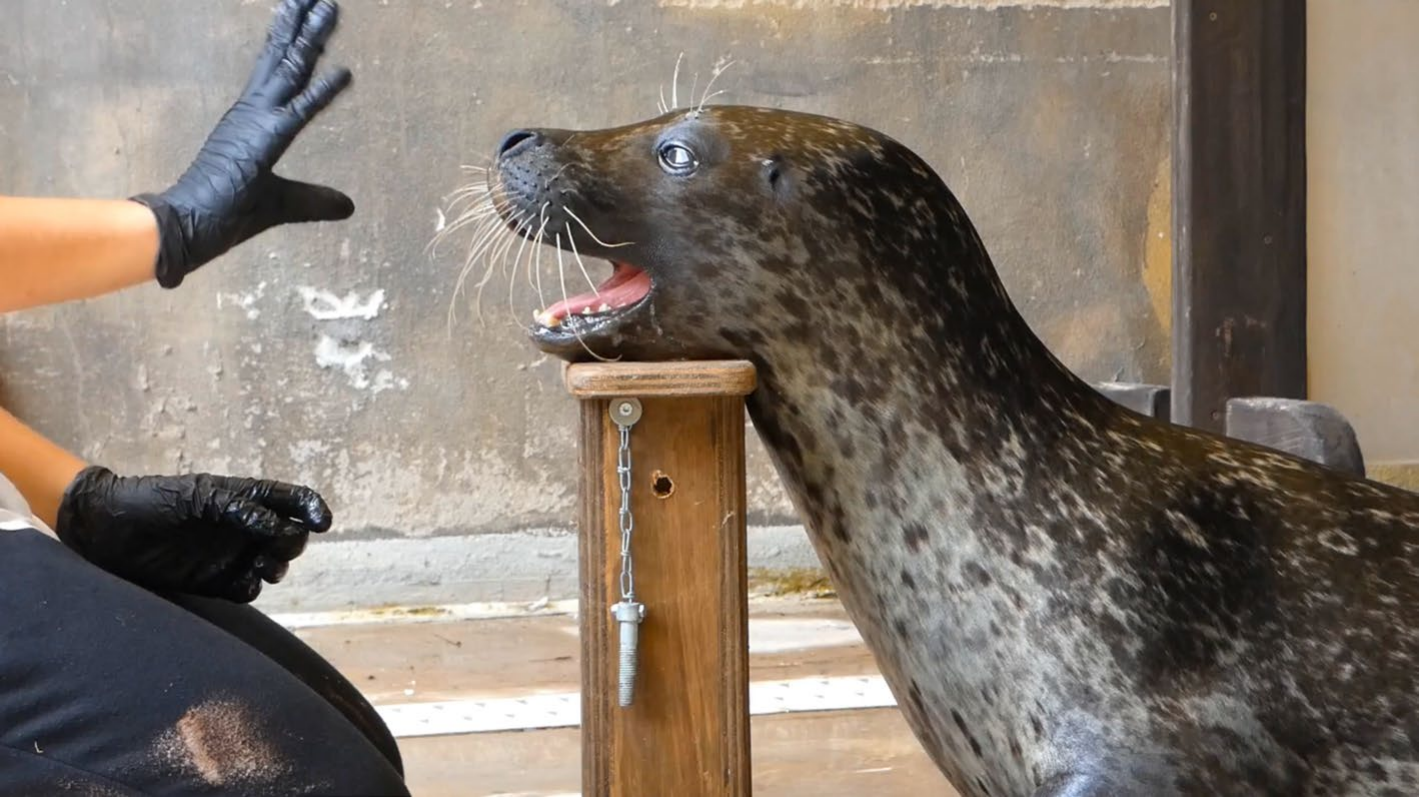
Positioning of the subject on the wooden platform during the experimental procedure (in this case, CD vocalization is vocally and gesturally prompted by the trainer); the jaw is passively resting on the platform allowing unobstructed vocalization

For the purposes of the current experiment, we introduced the following experimental setting, which consisted of two phases without intermediate breaks (Fig. 1a). In the first portion of each session, the trainer elicited the baseline (henceforth, BL) vocalization using the pre-existing corresponding discriminative vocal cue. During the second part of the session, the trainer prompted the new conditioned vocalization (henceforth, CD), which was elicited by both vocal, as “cantando”, and gestural cues, through the repeated opening and closing of the fingers of a hand (Figs. 1a and 2). For both phases, corresponding to the two vocalization types, the discriminative cue was repeated three to five times, adapting to the motivational state of the subject. In other words, using operant conditioning methods and positive food reinforcement, the trainers both continued eliciting the baseline, pre-existing vocalization mentioned above (BL), reinforcing it to maintain the original features, and trained the subject to produce a gradually modified version of the pre-existing vocalization to match a new conditioned vocal type (CD).

On the one hand, the baseline vocalization was positively reinforced if it matched the pre-existing acoustic features (no “wa” syllables) and would last at least 3 seconds. On the other hand, following the conditioning procedure proposed by Schusterman et al. [28], the trainers reinforced the conditioned vocalization when the subject would produce sounds that had “some vowel-like syllabic qualities” corresponding to [wa], that was emitted while the mouth was moving”. The vocalization was positively reinforced if both the production of the syllable “wa” and autonomous mouth opening and closing cycles were matched. This resulted in selective shaping of the conditioned vocalization to produce the “wa” sound in response to a specific cue, until it matched the repetition of the “wa-wa” sound. Specifically, they would reinforce it if it lasted at least 3 s, or if it included three complete cycles of the syllable “wa”. When the vocal type would match the cue, the subject was positively reinforced with a whistle and a fish reward would be provided: the two acoustic responses were thus placed under the control of different discriminative cues. Furthermore, the trainers would only reinforce the animal when its body remained completely still and the chin would rest firmly on the platform.

### Data collection

Data collection was conducted, under controlled and standardized conditions (see below), from October 2024 to March 2025, for a total of 54 sampled days in a period of 164 days (three days per week on average). Whenever possible, the subject participated in two daily training sessions, a morning session between 10:00 a.m. and 01:00 p.m. and an afternoon session between 2:00 p.m. and 4:00 p.m. Each session began with the trainer positioning the subject on the platform, a wooden support, 39 cm high, which ensured the standardized positioning of the body and the animal in space (Fig. 1a). The platform was placed in a designated area of the pinniped habitat, located in a transit zone regularly used by the animals to move between different pools. At a fixed distance of 262 cm from the platform, the researcher would record the prompted vocalization from a lateral view of the subject (Fig. 1a). This arrangement allowed continuous visual monitoring of the subject’s posture, with particular attention to the positioning of the chin on the platform, in order to avoid any mechanical interference on the vocal folds and larynx during the vocalizations. The researcher sat behind the tripod, operating synchronized audio recording equipment to document both vocal and behavioral data. The setup included a Sennheiser ME 66 directional microphone connected to a Zoom H6-BLACK digital recorder. Mono audio recordings were saved as WAV files, with a sampling rate of 96 kHz and a bit depth of 24 bits. Participation in training was never enforced: throughout the whole experimental season, the subject demonstrated consistent motivation and spontaneous willingness to engage in the sessions and his body weight varied consistently with typical physiological fluctuations observed in this species.

### Habituation period

Starting end of June 2024, before the data collection in the current experiment, the subject underwent a habituation period, during which it was progressively acclimated to the experimental setup, ensuring consistent exposure to the testing context and established procedural familiarity with the operant conditioning protocols described above. At the beginning of the habituation period, the animal was trained to use the platform, using a target whose tip the animal was trained to follow with its nose. The animal was first required to on-cue produce the baseline vocalization while using the platform. After a couple of weeks, the next step was the introduction of the new vocal and gestural cue (Fig. 2). During the first days of the introduction of the new cue, trainers associated a gentle touch of the subject’s nose and chin, encouraging the movement of the mouth while vocalizing, so as to stimulate the cycles of mouth closure and opening aimed at producing the target sound [28]. After about one month, the trainers had suspended all physical contact with the animal but maintained the vocal and gestural cues. Throughout the habituation period, the subject continued his typical training and husbandry behaviors, including the captured production of the baseline vocal behavior described in the previous section.

### Audio processing

#### Acoustic data annotation and pre-processing

Acoustic data processing was conducted using Praat (version 6.2.22 [47]). All recorded sessions were annotated on a TextGrid format. Specifically, the onset and offset of each vocalizations were marked, together with the required vocal type. Silent intervals or segments containing noise were labeled and excluded from subsequent acoustic analysis. Through an automated Praat script, the sound segments corresponding to the vocalizations were cut into separate WAV files. Following the methodology in Goncharova et al. [40], frequencies above 3000 Hz were filtered out, using Praat’s Filter (*stop Hann band*) with a smoothing factor of 50 Hz.

#### Formant contour extraction

Each extracted vocalization underwent the extraction of the first three formants contours (*To Formant (burg)* function; Praat [47]), defined as the dynamic trajectory of formant frequencies over time. Following Goncharova et al. [40], maximum formant frequency was set to 3000 Hz and the analysis window to 0.05 s; all other parameters were set to Praat’s default values. An example of extracted formant contours on the two vocal types, before any outlier removal, is visible in Supplementary Information (SI), Figure S1.

The above parameters were checked following the operational guidelines in [1]. Indeed, previous to the experiment, through six repeated measurements, we used a measuring tape to externally measure the distance between vocal folds and front teeth. These measures provided the subject’s estimated vocal tract length, corresponding to an average of 17 cm. This size matches average tract dimensions of adult harbor seals [48] and humans [49]. Importantly, a vocal tract size comparable to that of humans supports the reliability of Linear Predictive Coding-based formant estimation using standard analysis parameters [1]. Assuming a cylindrical vocal tract of this length, a simplified version of an actual seal vocal tract, we can apply the formula F_n_ = nc/4L, where n is the formant index, c is sound speed, L is the vocal tract length [1], for expected formant frequencies. This yields F1 at around 500 Hz for F1, 1500 Hz for F2 and 2500 Hz for F3. The vocal tract length reflected the appropriate number of poles for formant analysis (two poles per formant, six in our case [1]).

Furthermore, we proceeded to visually inspect around 5% (50 randomly sampled vocalizations) of the tentative extracted contours. The visual inspection of extracted formant contours allowed us to estimate tracking accuracy (overall: 93%; F1: ~98.5%; F2: ~91.2%; F3: ~95%). This also revealed the presence of poorly-tracked sections, especially at the start and end of the signal, consisting of deviations from the visible spectrogram formants, including biologically implausible frequency jumps between adjacent points on the contours. For this reason, visual estimation of the percentage of poorly tracked regions and the corresponding extent of the abovementioned frequency jumps guided the removal of statistical outliers. Specifically, we removed the upper and lower 2.5% values within each formant distribution from the sample (for a total of 5%). Furthermore, we calculated frequency transitions - quantified as the absolute difference between adjacent points of the formant contours (i.e. within 0.0125 s; later in the manuscript, Modulation Depth) – and, when these fell within the top 5% of the most abrupt frequency transitions, the second point of the couple producing the unlikely frequency jump was excluded. The removal of these outliers excluded frequency jumps that were higher than ± 47.529 Hz for F1, ± 98.218 Hz for F2, ± 90.672 Hz for F3. Outlier removal was both visually (SI - Figure S2) and statistically (SI - Table S2) inspected, by checking that the central tendency of the data (e.g., median) remained stable, impacting only tails’ reduction, a proxy for successful mitigation of extreme values without distorting the overall data structure. Visual inspections confirmed the improved distribution symmetry and range, and key summary statistics showed appropriate changes consistent with outlier reduction. To further validate our approach, we selected 20 well-tracked files (mean estimated tracking error: 2.70%, [1.13–4.12%]) and simulated an extreme case of tracking error scenario, where each formant could be misassigned ( e.g., F3 tracked as F1). Note that this type of error is rare in Praat when extraction settings are optimized. To simulate the errors, we shuffled 2–10% of observations within time points. For each level, we computed summary statistics and quantified the average error as the percentage deviation from the original mean. These metrics were calculated both before and after the paper’s outlier removal procedure (± 2.5% tails, and > 5% contour jumps). As shown in the plots and table (S3a-b), deviations from the original, correct values were minimal (< 1% after outlier removal), supporting the robustness of our approach.

### Measures of formant modulation

#### Modulation coefficients of variation

First, we calculated the coefficient of variation (CV; e.g [50]) , as

$${\text{CV}}\,=\,\sigma /\mu $$

where σ is standard deviation of the formant contour and µ is the mean value of the formant contour, for each recorded vocalization and for each of the three formants separately. The CV captures the overall variability in formant frequency (Hz) emission, providing information on the acoustic stability or instability of the produced sounds. A higher variability in the formant contour corresponds to higher CVs, indicating greater within-vocalization modulation in formant values.

#### Modulation depth

Next, we calculated a measure of the formant modulation slope per time unit (e.g [51]). Within each formant (F1, F2, F3), within a single vocalization, we calculated

$${\text{Modulation Depth }}={\text{ }}\left| {{\text{ }}{{\text{f}}_{{\text{k}}+{\text{1}}}} - {\text{ }}{{\text{f}}_{\text{k}}}} \right|$$

Where f_k_ and f_k+1_ are adjacent points of a formant contour f_1_ ,…, f_k_ ,…,f_n_ (one point every 0.05 s). Henceforth in the manuscript, this measure is referred to as Modulation Depth. A greater Modulation Depth, corresponding to greater frequency shift per unit time, reflects greater modulation of formants.

#### Modulation spectral entropy

A signal can show more or fewer shifts in formant modulation per unit time, but whether these shifts are temporally predictable or randomly organized needs to be assessed through specific measures. To delve into this aspect, we first extracted the dominant formant contour periodicity, in order to identify the appropriate window length to calculate the Spectral Entropy on. To this aim, we extracted the range of dominant formant oscillations through wavelet transform (*analyze.wavelet()*, function in *WaveletComp* R package, version 1.2 [52]). We then divided the signal into sliding windows composed of 40 adjacent points in the contour, corresponding to a time window of 0.5 s (formant contour time window*number of points), in order to ensure the capture of at least one full cycle of periodicity in the modulation of the formant contour. We then normalized power spectral densities (PSDs) into a probability distribution per each vocalization, so all values sum to 1. We set the step size between adjacent windows as 20 points (0.25 s), corresponding to 50% overlap between adjacent windows, in order to avoid sharp Entropy values jumps or fluctuations between windows and to smooth their transitions, giving a more stable and interpretable output. Finally we extracted Shannon Entropy [53] for each window. This approach provided a dataset of one value of Spectral Entropy every consecutive 40 points of the formant contour. An unstructured variation in the formant contour would correspond to a complex spectrum, showing energy at many frequencies, and thus higher Entropy. A predictable, sinusoidal modulation in a formant contour, as expected from the repeated alternation of vowels, would correspond to a spectrum which shows few discrete peaks spread across non-zero frequencies, corresponding to lower Entropy values.

### Statistical analysis

#### Formant contour values

Statistical analyses were performed in R [version 4.4.2, 54]. We first tested whether the overall covered range of formant frequencies varies throughout the experiment and between different vocal types through linear mixed models (LMM). As, overall, the covered frequencies would show a trimodal distribution (one local maximum per each formant), we built three separate full models, one for each formant F1, F2, and F3, using the *glmmTMB* package in R [version 1.1.11, 55]. Each of them included the formant values as the response variable, and vocal type (BL or CD) and time (as day number, i.e., an increasing integer corresponding to the date of recording) as explanatory variables. The interaction between the fixed term was included and retained only when significant (F1 and F3). The formant contours (response variables) followed a symmetrical distribution (*fitdistrplus* R package version 1.2.2 [56], ; estimated skewness: -0.007, estimated kurtosis: 2.610), but they showed heavy tails on lower and upper values. Through the *bestNormalize* package in R (version 1.9.1 [57], ; Estimation method: Out-of-sample via CV with 10 folds and 5 repeats), they were thus transformed with an Ordered Quantile normalizing transformation (*orderNorm*). The models were then fitted on a Gaussian family. A random effect for the specific vocalization code was included to account for baseline differences across groups [e.g., recording sessions coinciding with variable motivational states, 58]. For each model, a visual check confirmed an appropriate distribution of residuals, ensuring the model’s assumptions were met, through the package *performance* in R [version 0.14.0, 59]. To test the significance of the full models, we compared them against a null model only comprising the random factors (likelihood ratio test - Anova with ‘Chisq’ argument [60]). The summary of the full model, obtained via the summary() function in R, provided estimates, standard errors, test statistics (z), and p-values for each main effect and interaction term. Effect plots were produced through the *plot_model()* function (*sjPlot* R package version 2.8.17 [61]). When an interaction term was present, slopes per vocal type, representing the effect of the predictor within each group, were extracted through *emtrends* function (*emmeans* R package version 1.11.1 [62], ; default p-adjustments through Tukey method).

#### Formant contours variation

For each vocalization, and each formant separately (F1, F2, F3), we calculated the coefficient of variation (CV). We tested the trends of CVs per vocal type and throughout the experiment using a LMM. The response variable (CVs) followed a left skewed distribution (min: 0.012, max: 0.186, median: 0.061, mean: 0.065, estimated sd: 0.023, estimated skewness: 0.659, estimated kurtosis: 3.131). It was thus transformed through the *bestNormalize* R (version 1.9.1 [57], ; Estimation method: Out-of-sample via CV with 10 folds and 5 repeats) to better approximate normality (family = *Gaussian()*). The fixed factors included, in interaction, the formant, the vocal type and the day number. As we calculated one CV per vocalization and formant, we used the identification code of each experimental session as random intercepts and checked for singularities. We visually inspected the residual distribution and uniformity [*performance* R package version 0.14.0, 59]. The summary of the full model, obtained via the *summary()* function in R, provided estimates, standard errors, test statistics (z), and p-values for each main effect and interaction term. Post-hoc comparisons and slopes per vocal type were obtained through the *emtrends* function (*emmeans* R package version 1.11.1 [62], ; default p-adjustments through Tukey method). An effect plot of the model was produced through the *plot_model()* function (*sjPlot* R package version 2.8.17 [61]).

#### Formant modulation depth

To analyze the formant Modulation Depth, for F1, F2, and F3 simultaneously, a linear mixed model (LMM) was fitted using the glmmTMB package in R. Because the distribution of the response variable (Modulation Depth) was strongly right skewed and strictly positive (*fitdistrplus* R package version 1.2.2 [56], ; min: 0.000 Hz; max: 118.218 Hz; median: 18.247 Hz; mean: 25.204 Hz; estimated sd: 22.9711 Hz; estimated skewness: 1.347, estimated kurtosis: 4.541) it was transformed through *bestNormalize* package in R (version 1.9.1 [57], ; Estimation method: Out-of-sample via CV with 10 folds and 5 repeats) to better approximate normality (family = *Gaussian()*). The full model included the interaction between formant, vocal type, and day number, with a random intercept for vocalization identification code to account for baseline differences across groups. For each model, a visual check confirmed an appropriate distribution of residuals, ensuring the model’s assumptions were met. The full model was then compared against a null model only comprising the random intercepts (likelihood ratio test - Anova with ‘Chisq’ argument [60]), to test whether the fixed factors of interest significantly improved the model’s ability to explain the data. The summary of the full model, obtained via the *summary()* function in R, provided estimates, standard errors, test statistics (z), and p-values for each main effect and interaction term. Post-hoc comparisons and slopes per vocal type were obtained through the *emtrends* function (*emmeans* R package version 1.11.1 [62], ; default p-adjustments through Tukey method). An effect plot of the model was produced through the *plot_model()* function (*sjPlot* R package version 2.8.17 [61]).

#### Formant contours predictability: spectral entropy

For each vocalization, the formant contour predictability per time unit was determined through Spectral Entropy across the first three formant (F1, F2, F3) and analyzed using LMMs. The response variable (Spectral Entropy) followed a left skewed distribution (min: 1.756, max: 4.254, median: 3.427, mean: 3.341, estimated sd: 0.464, estimated skewness: -0.676, estimated kurtosis: 2.847). For this reason, it was transformed through the *bestNormalize* R (version 1.9.1 [57], ; Estimation method: Out-of-sample via CV with 10 folds and 5 repeats) to better approximate normality (family = *Gaussian()*). The fixed factors included, in interaction, the formant, the vocal type and the day number. As shorter vocalizations only included one single value of SE, we didn’t use the vocalization code as random intercepts, which would result in a singular fit, but we included the identification code of each experimental session instead. As for previous models, we visually checked the residual distribution and uniformity [*performance* R package version 0.14.0, 59]. The summary of the full model, obtained via the *summary()* function in R, provided estimates, standard errors, test statistics (z), and p-values for each main effect and interaction term. Post-hoc comparisons and slopes per vocal type were obtained through the *emtrends* function (*emmeans* R package version 1.11.1 [62], ; default p-adjustments through Tukey method). An effect plot of the model was produced through the *plot_model()* function (*sjPlot* R package version 2.8.17 [61]).

All calculated acoustic variables and all the variables included in the statistical analyses are summarized in SI, Table S4.

#### Formant modulation-based classification of vocal types over time

Through the previous analysis steps, we proposed three measures beyond formant frequencies (Coefficients of Variation, Modulation Depth and Spectral Entropy) to measure formant modulation, and its course throughout the experiment. As a last step of the analyses, we wanted to assess how the two vocal types diverged acoustically over the experiment in these three measures. In other words, we evaluated their separability in the acoustic space, at the start vs. at the end of the experiment, on the basis of the formant modulation measures which, in earlier stages of analysis, showed significantly divergent trends over the course of the experiment. This was the case with respect to F1 and F3 modulation measures. This was achieved using a well-established two-step approach: based on the previously described formant modulation measures, first, dimensionality reduction was performed via Uniform Manifold Approximation and Projection (UMAP), a non-linear dimensional reduction technique particularly effective for data visualization, followed by classification using a Random Forest machine learning algorithm [63]. The integration of dimensional reduction before performing classificatory algorithms facilitates visualization, reduces computational cost, mitigates overfitting, and improves model performance. As far as we are aware, no prior work has applied dimensionality reduction and classification using formant modulation.

First of all, we split our dataset between observations referring to the start (1st quartile of the sampled days) vs. the end of the experiment (4th quartile of the sampled days). However, the CVs were calculated at the vocalization level, Modulation Depth was at the scale of the dyad of formant contour adjacent points, and Spectral Entropy was expressed by one value every 40 points in the contours. For this reason, we summarized these measures, for F1 and F3 separately, as follows: we calculated the distribution quartiles for the Modulation Depth and Entropy measures, for each vocalization. Coefficients of variation (CV) for F1 and F3 were included to quantify relative variability for each file and were already calculated at the file level. Through this approach, we were able to preserve the complex structure of our data, making it comparable at the level of vocalization. Only complete cases were included in the UMAP analyses, providing a total of 304 vocalizations (160 at the start and 144 at the end of the experiment; 148 baseline vocalizations and 156 conditioned ones).

For each vocalization, one value of each of the 18 input variables was used in the UMAP. As detailed above, these included, for both F1 and F3, the coefficient of variation, the 1st through 4th quartiles of Modulation Depth, and the 1st through 4th quartiles of Spectral Entropy, resulting in 9 variables per formant. Two separate UMAPs were performed, one run on the vocalizations recorded at start and one on the vocalizations recorded at the end of the experiment. To visualize structure in the acoustic feature space, uniform manifold approximation and projection (UMAP; [64]) was applied to the dataset. All acoustic predictors were included in the embedding. UMAP was run using the *umap* R package (version 0.2.10.0; [65]) with a fixed random seed to ensure reproducibility. The resulting low-dimensional representation provided two-dimensional coordinates (V1 and V2) for each observation. The UMAP coordinates were extracted into a data frame and merged with vocal type labels, allowing later visualization and classification.

UMAP coordinates, labelled per vocal type, were included in the training phase of the two classification models, one for the start and one for the end of the experiment. Training (*randomForest()*) was performed on randomly sampled 70% of the data, vocal type was used as a response variable for the classification and 800 decision trees were specified. The training phase provided an OOB estimate of error rate and a confusion matrix. Through the *importance()* function, we were able to assess the relevance of each PC in the classification training. In particular, *MeanDecreaseAccuracy* quantifies the impact of each vocal type on model prediction accuracy and *MeanDecreaseGini* quantifies the contribution of each vocal type to node impurity reduction. Plotting the model error rate vs. the number of trees, we assessed whether an adequate number of trees has been grown by observing if the model error rate reaches a plateau, indicating stability in performance. We then moved to the testing phase (*predict()* function on the remaining 30% of the data). We plotted the Receiver Operating Characteristics (ROC; *ROCR* R package version 1.0.11 [66]), for both models and extracted the Area Under Curve (AUC): this allowed us to evaluate and compare the predictive performance of both models by measuring their ability to discriminate between vocal type classes. We performed both models on the whole dataset and plotted the real vs. predicted vocal type for each data point, for both models.

The last step of the analyses consisted in the quantitative models comparison (start vs. end of the experimental period). ROC curves, and corresponding AUC, are commonly used for model comparison across datasets, as they are not directly influenced by the proportion of positive and negative cases [67]. We thus performed a Delong test (*roc.test()* function, *pROC* R package version 1.18.5 [68]), to compare the AUC curves of the two models. Delong test statistically compares the AUCs of two classifiers, to test whether the difference in AUC between two models is statistically significant.

On top of AUC, of the several performance measures we extracted through *confusionMatrix()* function (*caret* R package version 7.0.1 [69]), only the following are suitable for comparing model performance across different datasets, as they are not directly influenced by class prevalence. In other words, these metrics evaluate a model’s ability to distinguish between classes regardless of how frequent each class is in the data. These are Balanced Accuracy (which accounts for class imbalance by averaging recall/sensitivity of each class [70]), Sensitivity (or Recall, i.e., the ability to detect BL correctly, the true positives), Specificity (i.e., the ability to detect CD correctly, the true negative [71]).

## Results

### Collected data

A total of 1095 vocalizations were collected. In particular, over the 54 number of sampled days during an overall period of 164 days, we recorded 455 baseline and 640 conditioned vocalizations. We excluded 11 vocalizations from the sample since the positioning of the animal on the platform was not correct.

### F1 and F3 contour values differ between vocal types

**Fig. 3:**
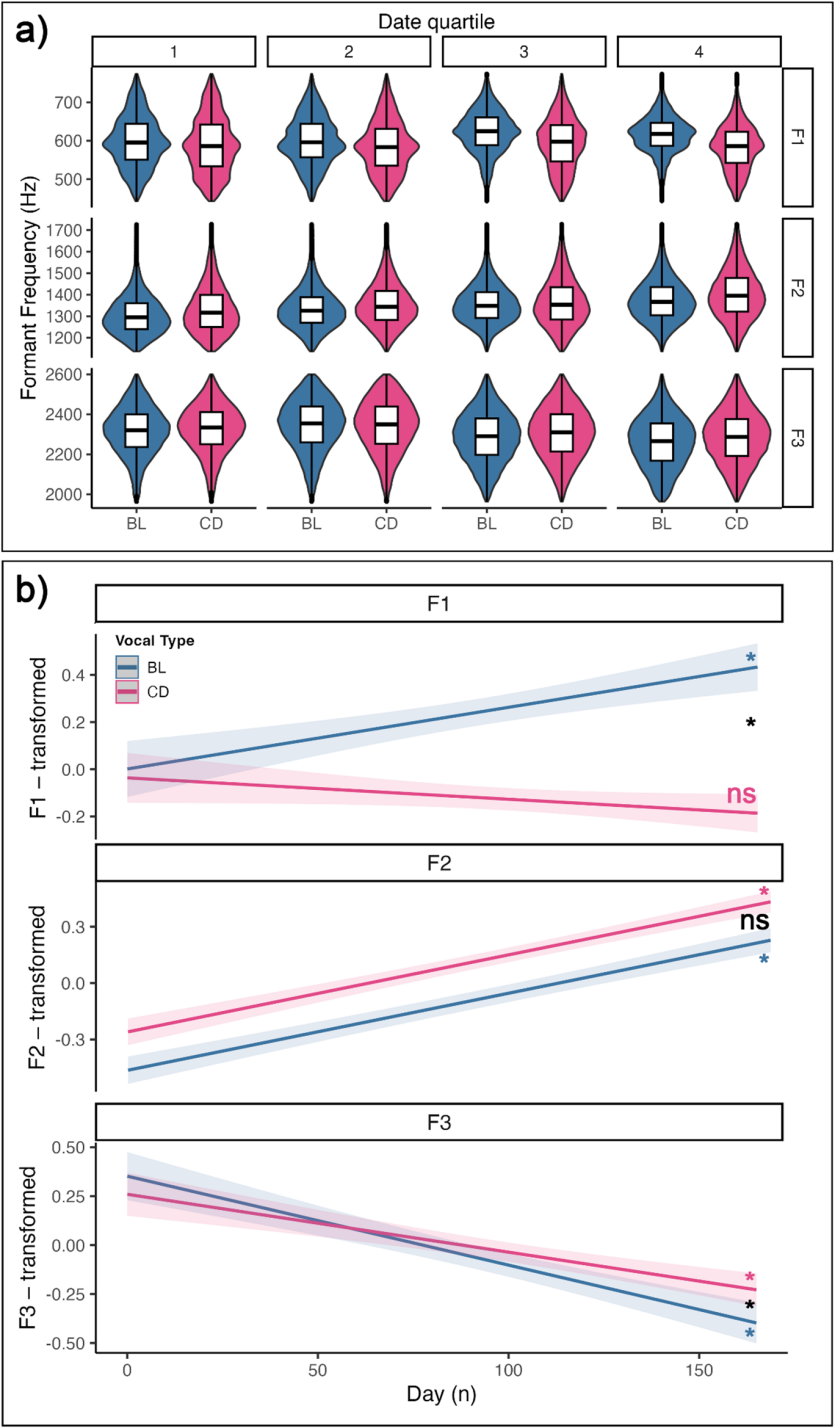
**a** Violin plots combined with boxplots displaying the distribution of the formant contour values, per formant and across the experiment. The date quartiles refer to the distribution quartiles of the experiment sampled days. Violin plots display the density distribution, i.e., a smoothed outline of the histogram of values, across different groups. Boxplots show the median (horizontal line), interquartile range (box), and 1.5×interquartile range whiskers across different groups; points beyond the whiskers represent potential outliers. **b** Effect plots generated from the LMMs testing for the effect of time (day number) and vocal type on the formant values. The shaded area around the regression lines represents 95% confidence intervals. Slope significance is marked near each line in vocal type colors (* = *p* < 0.05; ns = not significant); significance of slope difference is shown in black

Descriptive statistics of the formant contour distributions, before and after the removal of outliers, can be found in S2. We divided the experimental days into quartiles: Fig. 3a displays the distribution of the formant contour values throughout the experiment (per quartile of sampling period and per formant). Tables in S5 report the comparison of full vs. null models, the summary of the full models, and the slopes per vocal type. Estimated slopes reflect the trends in formant values for each vocal type across the experiment. The effect plots of the models in Fig. 3b visually represent the trends of formant values, per vocal type, throughout the experiment. The significance of the slope per vocal type, and their comparison, are marked with asterisks when significant.

All full models showed a significantly better fit than their corresponding null. F1 values didn’t differ between vocal types at the beginning of the experiment (Estimate = -0.038, z = -0.466; *p* = 0.641; S5a). However, a significant interaction between vocal type and day number (Estimate = -0.004, *z* = -4.628, *p* < 0.001) indicated that the trajectory of F1 differed between vocal types throughout the experiment (S5a): only BL vocalization significantly changed with day number (BL slope = -0.004, 95 CI% [0.001, 0.004]; S5b).

Day number had a significant positive main effect on F2 values (Estimate = 0.004, z = 13.530, *p* < 0.001; S5c). At the start of the experiment, F2 was higher in CD compared to BL (Estimate = 0.204, *z* = 6.600, *p* < 0.001; S5c). No interaction term was included for this model, as it was not significant, indicating similar slopes between vocal types.

F3 values were similar between vocal types at the start of the experiment (Estimate = -0.093, z = -1.101, *p* = 0.271; S5d). However, a significant interaction between vocal type and day number (Estimate = 0.002, *z* = 2.010, *p* = 0.045; S5d) indicated different F3 trajectories between vocal types. The change throughout the experiment was significant and negative for both vocal types, and steeper for BL (BL Estimate: -0.004, 95% CI [-0.006, -0.003]; CD Estimate = -0.003, 95% CI[-0.006, -0.003]; S5e).

To sum-up, the formant trends differed between vocal types in F1 and F3, but not F2. While in F1 the two vocal types showed opposite trajectories, in F3 they both showed a significant reduction in frequency as the experiment proceeded.

### Modulation measures

#### Formant contours coefficients of variation (CVs): F1 and F2 within-contour variations differ between vocal types

**Fig. 4:**
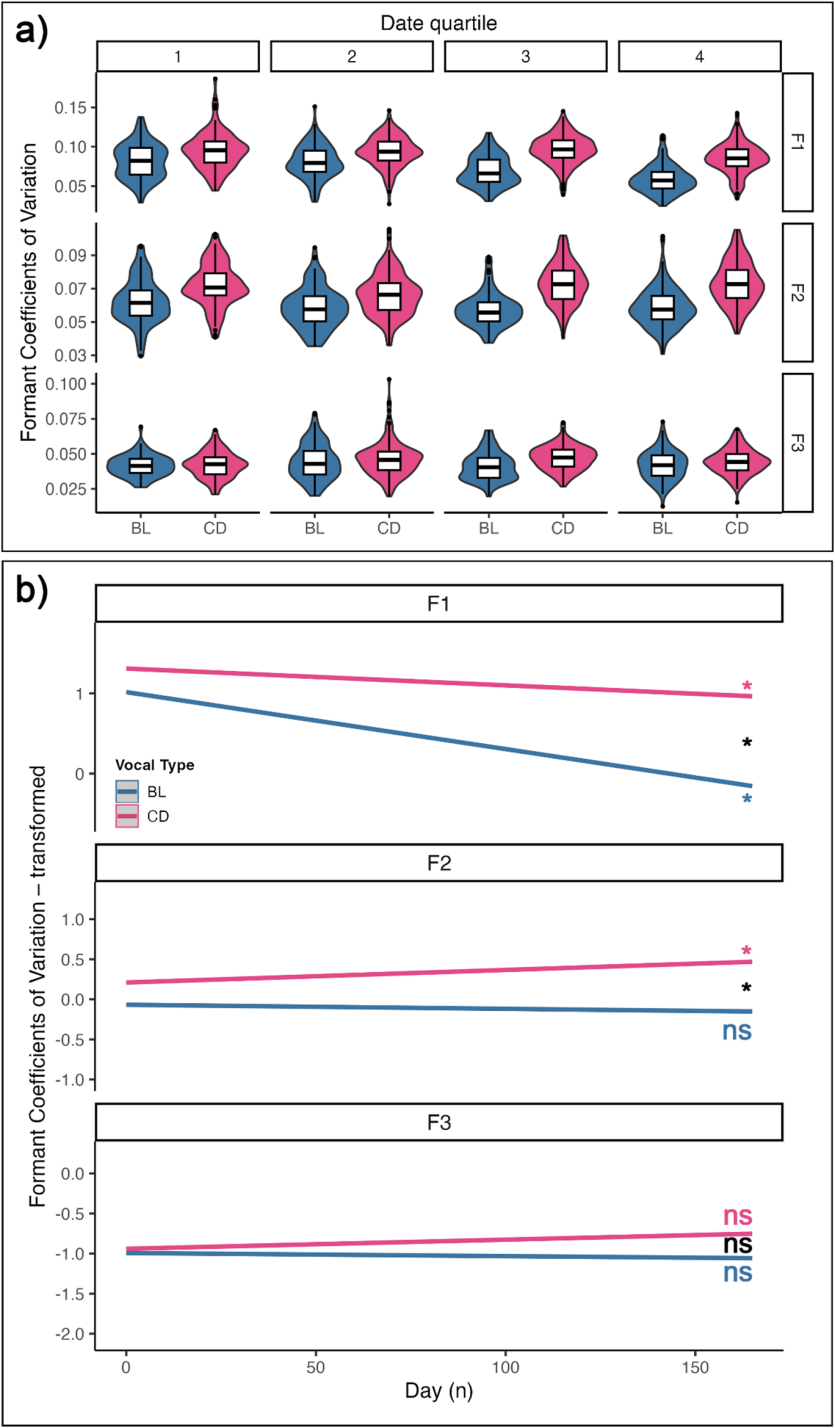
**a** Violin plots combined with boxplots displaying the distribution of the Coefficients of Variation (CVs), per formant and across the experiment. The date quartiles refer to the distribution quartiles of the experiment sampled days. Violin plots display the density distribution across different groups. Boxplots show the median (horizontal line), interquartile range (box), and 1.5×IQR whiskers across different groups; points beyond the whiskers represent potential outliers. **b** Effect plots generated from the LMM testing for the effect of formant, time (day number) and vocal type on the CV values. The shaded area around the regression lines represent 95% confidence intervals. Slope significance is marked near each line in vocal type colors (* = *p* < 0.05; ns = not significant); significance of slope difference is shown in black

**Fig. 5:**
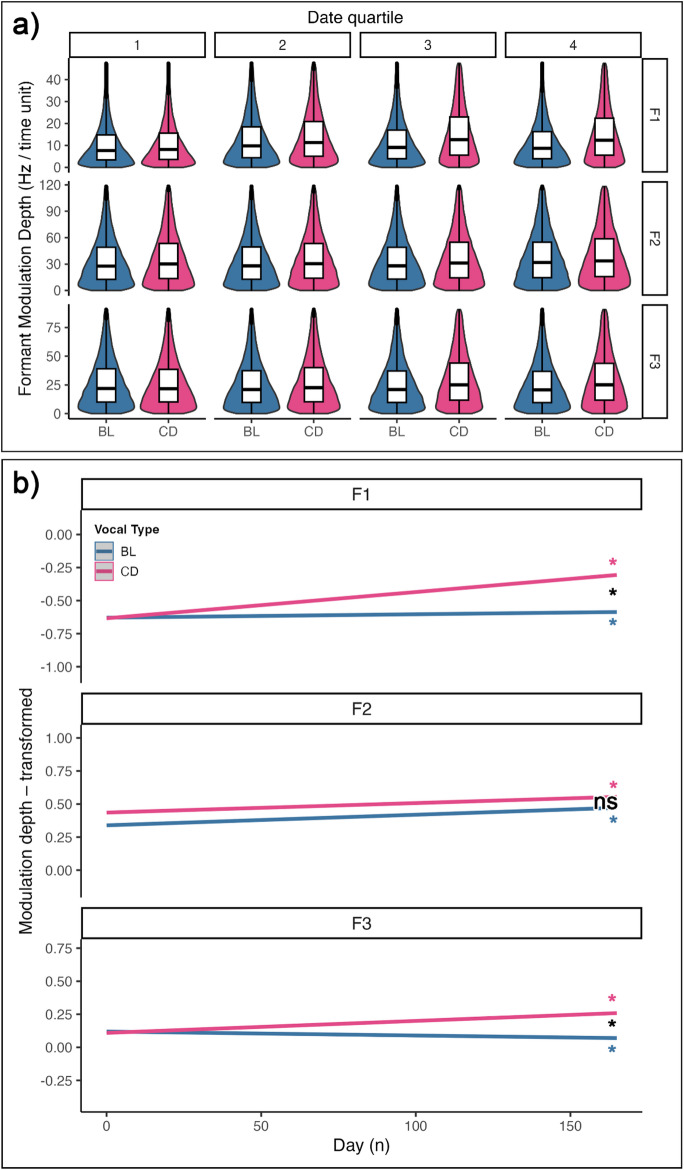
**a** Violin plots combined with boxplots displaying the distribution of the formant Modulation Depth values, per formant and across the experiment. The date quartiles refer to the distribution quartiles of the experiment sampled days. Violin plots display the density distribution across different groups. Boxplots show the median (horizontal line), interquartile range (box), and 1.5×IQR whiskers across different groups; points beyond the whiskers represent potential outliers. **b** Effect plots generated from the LMM testing for the effect of formant, time (day number) and vocal type on the formant Modulation Depth values. The shaded area around the regression lines represent 95% confidence intervals. Slope significance is marked near each line in vocal type colors (* = *p* < 0.05; ns = not significant); significance of slope difference is shown in black

To quantify the variation of formant contours in each vocal type, we extracted one CV for every vocalization and every formant (F1, F2, F3). Figure 4a shows the distribution of CVs, by quartile of days of the experiment. Tables S6 report the comparison between full and null models, the summary of the full model, and the estimated slopes, which represent the trends in formant values for each vocal type across the experiment. The effect plots of the models in Fig. 5b visually depict these effects. The significance of the slope per vocal type, and their comparison, are marked with asterisks when significant.

The model revealed that F1 decreased for both vocal types throughout the experiment (BL Estimate = -0.0071, 95% CI [-0.0084, -0.0058]; CD Estimate = -0.0021, [-0.0033, -0.0009]; S6b). Importantly, BL decreased more than CD (BL vs. CD - Estimate = -0.0050, SE = 0.0008, t-ratio = -6.320, *p* < 0.0001; S6c). However, F2 increased in CD but not in BL (CD estimate = 0.0016, 95% CI [0.0004, 0.0027]; S6b). No effect of day number was registered for F3.

#### Formant modulation depth: F1 and F3 of the conditioned vocalization increase their frequency shifts over time

We quantified Modulation Depth as the absolute value of the difference between adjacent points of the formant contours. Figure 5a shows the distribution of Modulation Depth throughout the experiment (quartiles of the data collection period) for each formant. We tested whether the amount and trend of Modulation Depth, as the training progresses, would change between vocal types. Tables **S7** report the comparison between full and null models, the summary of the full model, and the estimated slopes, which represent the trends in formant modulation depth for each vocal type across the experiment. The effect plots of the models in Fig. 5b visually depict the trend of formant modulation depth per vocal type, throughout the experiment. The significance of the slope per vocal type, and their comparison, are marked with asterisks when significant.

The the two vocal types did not differ in Modulation Depth at the start of the experiment (Estimate = − 0.005, *z* = − 0.38, *p* = 0.701; S7a). As most of the two- and three-ways interactions were significant (S7a), we performed post-hoc comparisons to unpack specific interactions. For all three formants and both vocal types, the Modulation Depth would significantly changed throughout the experiment. In particular, all slopes significantly increased (F1: BL estimate = 0.0003, 95% CI [0.0010, 0.0040]; CD estimate = 0.0020, 95% CI [0.0018, 0.0021]; F2: BL estimate = 0.0008, 95% CI [0.0006, 0.0010]; CD estimate = 0.0007, 95% CI [0.0006, 0.0009]; CD estimate = 0.0009, 95% CI [0.0008, 0.0011]; S7b), exempted for the modulation of F3 in the BL vocalization (F3: BL estimate = -0.00031, 95% CI [-0.0005, -0.0001]; S6b). Crucially, for F1 and F3, CD showed a significantly steeper, positive slope than BL (BL vs. CD - F1: Estimate = − 0.002, *t* = − 14.684, *p* < 0.001; F3: Estimate = − 0.001, *t* = − 10.198, *p* < 0.001; **S7c**).

To sum-up, at the beginning of the experiment the two vocal types showed similar values of Modulation Depth, but, throughout the experiment, F1 and F3 increased significantly more in CD than in BL.

#### Predictability of the formant contour: all formants show increased predictability in the formant contours of the conditioned vocalization

**Fig. 6:**
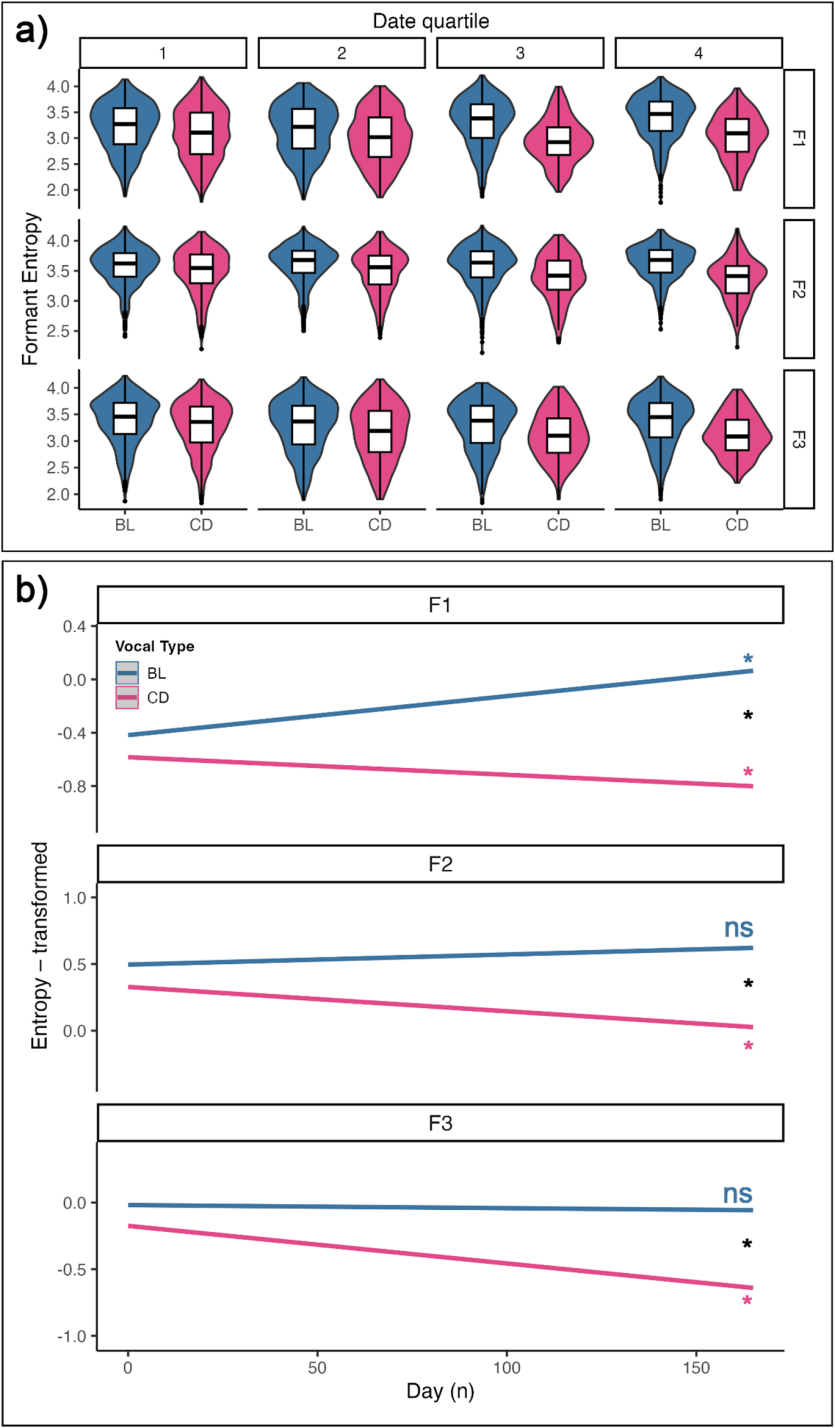
**a** Violin plots combined with boxplots displaying the distribution of the formant contour Spectral Entropy values, per formant and across the experiment. The date quartiles refer to the distribution quartiles of the experiment sampled days. Violin plots display the density distribution across different groups. Boxplots show the median (horizontal line), interquartile range (box), and 1.5×IQR whiskers across different groups; points beyond the whiskers represent potential outliers. **b** Effect plots generated from the LMM testing for the effect of formant, time (day number) and vocal type on the formant contour Spectral Entropy. The shaded area around the regression lines represent 95% confidence intervals. Slope significance is marked near each line in vocal type colors (* = *p* < 0.05; ns = not significant); significance of slope difference is shown in black

To quantify the predictability of the formant contour modulations, we quantified the spectral entropy of the contours on 0.5 s segments of the vocalizations. This time window choice was validated by the extraction of the dominant frequency of the contours through Wavelet transform, which gave a peak overall frequency at 0.25 s (4 Hz), for both vocal types and for the three formants separately. The dominant frequencies across vocalizations overall spanned between 0 and 0.5 s, making the 0.5 s time window appropriate to capture at least one cycle of oscillation. The distributions of Spectral Entropy values are visible in Fig. 6a (per quartiles of the data collection period).

Similarly to what was done for the Modulation Depth values, we built a model to test whether the amount and trend of signal structuring, as the training progresses, would change between vocal types. Tables S8 report the comparison between full vs. null models, the summary of the full model and the slopes per group, which represent the trends in formant entropy for each vocal type across the experiment. The effect plots of the models in Fig. 6b visually depict the trend of formant entropy per vocal type, throughout the experiment. The significance of the slope per vocal type, and their comparison, are marked with asterisks when significant.

Most of the two- and three-way interactions in the model were significant (S8a). Indeed, both BL and CD showed a change in F1 entropy values throughout the experiment (BL slope = 0.003, 95% CI [0.002, 0.004]; CD slope = -0.001, 95% CI [-0.003, -0.001]; S8b). Crucially, F2 and F3 only changed for CD (F2: CD slope =-0.002, 95% CI [-0.003, -0.001]; F3: CD slope = -0.002 95%, CI [-0.005, -0.002]; S7b). For all three formants, CD showed a significantly higher decrease in Spectral Entropy than BL vocalizations (F1: slope = -0.001, 95% CI [-0.003, -0.001]; F2: slope = -0.002, 95% CI [-0.003, -0.001]; F3: slope = -0.003, 95% CI [-0.005, -0.002]; S8c).

Overall, these results show that CD shows lower Spectral Entropy, i.e., higher predictability of the formant contour, throughout the whole experiment, with a significant negative slope. BL increased in F1 entropy but remained stable in F2 and F3 throughout the experiment.

### Vocal types classification across the experiment

The quantification of specific formant modulation measures and their trend over the course of training revealed a gradual divergence between the two vocal types as the experiment proceeded. To validate the differentiation between the two vocal types, we quantified the performance of classification algorithms at the start vs. at the end of the experiment only on the basis of F1 and F3 modulation measures (Coefficients of Variation, Modulation Depth, Spectral Entropy). Dimensional reduction through UMAP was applied first to the modulation measures of the vocalizations corresponding to the start and then to the end of the experiment. Overall, our approach revealed that, through the calculated formant modulation measures, the two vocal types are significantly easier to discriminate at the end of the experiment.

**Fig. 7:**
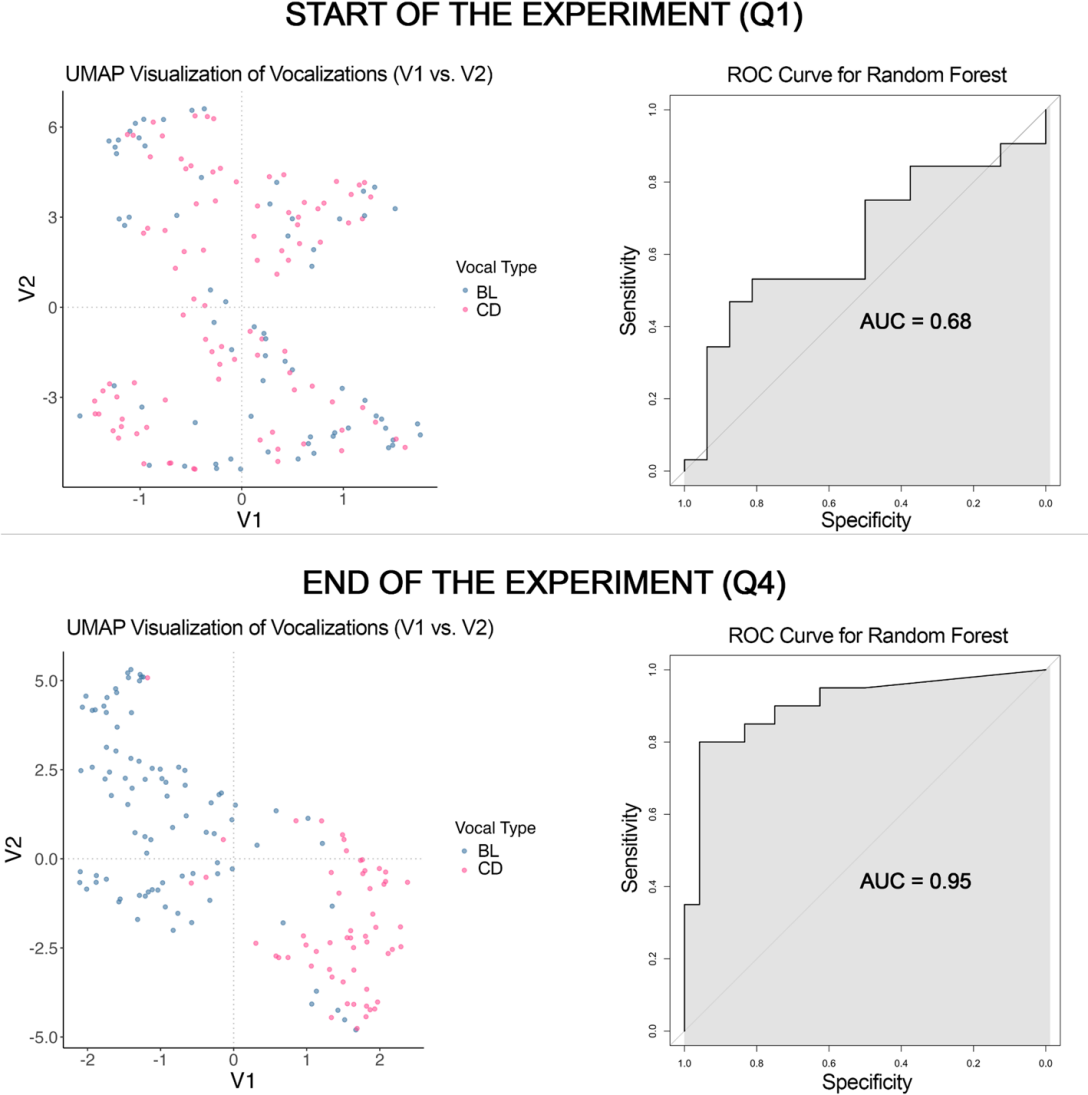
On the left, scatter plots display the distribution of the vocalizations in the acoustic space (on the basis of the first two PCs), per vocal type (BL in blue, CD in pink). On the right, the ROC plots show the tradeoff between specificity and sensitivity for the two Random Forests classification models testing phase (start vs. end of the experiment): classifiers with random performance show a diagonal line from (0, 0) to (1, 1) which corresponds to a baseline of ROC. A ROC curve provides a performance measure called the Area under the ROC curve (AUC) score. AUC is 0.5 for random and 1.0 for perfect classifiers [72]

After applying dimensional reduction through UMAP, we obtained, for each vocalization file, two coordinates in the acoustic space (V1, V2). As can be appreciated from the scatter plots representing the distribution of data points based on the UMAP components (V1, V2) in Fig. 7, the acoustic space occupied by the two vocal types is significantly more overlapped at the beginning than at the end of the experiment. On those dimensions, we trained the two Random Forest classification models on 70% of all the data. This provided a close-to-chance Out-of-Bag (OOB) error at the start in the model at the start of the experiment (49%) and a very low rate at the end (8%; SI, table S9). For both models, we obtained the class error for each vocal type: 54% for BL and 45% for CD vocalizations at the start of the experiment; only 8% for both BL and CD vocalization at the end of the experiment (SI, table S9). The testing phase of the two models, performed on the remaining 30% of the data, returned an Area Under Curve (AUC) of 0.64 at the start of the experiment and 0.90 at the end (Fig. 7). In line with that, based on Delong’s Test, performance was significantly higher in the model at the end of the experiment (D = -2.746, df = 74.762, *p* < 0.01): even though both models performed better than chance, already indicating a separation from the start of the experiment, the model referring to the end of the experiment consistently outperformed the first across calculated performance metrics. Balanced Accuracy, Sensitivity and Specificity are prevalence-independent and can be compared across models: all the performance measures were higher values on the classification model performed on vocalizations emitted at the end of the experiment (**S9**).

## Discussion

### Discussion of individual findings

We tested a harbor seal’s capacity for trained formant modulation. While maintaining cued baseline vocalization (BL), we trained the seal to produce, in response to a new cue, a conditioned vocalization (CD) ex novo by opening and closing its upper vocal tract. We compared this CD to the BL over the course of the experiment. Unlike the BL vocal type, the CD was trained as “phonating while opening and closing the mouth”: this should result in CD showing increased formant modulation over the experiment. Our results support this hypothesis along 4 lines of evidence based on measurements on the first three formant frequencies. We dissect these lines of evidence in the following 4 paragraphs.

The introduction of new articulatory movements in the training routine was expected to impact formant values in CD vocalizations. Indeed, F2 and F3 changed over the course of the experiment for CD. At the end of the experiment, F1 differed between vocal types, and so did F3. Overall, most formant values differed between vocal types and also over time; i.e., the average value of F1 (or F3) differed between the start and the end of the experiment. This suggests that the training of mouth movements while phonating indeed resulted in learnt overall changes in formants. Interestingly, the interaction term in the model for F1 contour was significant: F1 of BL, but not CD vocalizations, changed throughout the experiment. Because both vocal types were reinforced, the contingency may have encouraged *any* change that increased reward likelihood, also in BL. That would promote a formant values shift of BL, in parallel to a focused, target-directed change in CD formant *modulation*, but not the overall formant values. Overall, the increase of both F1 and F2 in BL suggests a reduction of the inter-formant dispersion, i.e., the spacing between adjacent formants. In contrast, F1 remained stable but F2 increased in CD, suggesting a distancing of F1–F2, and a spread of vowel space, in line with stronger articulatory contrasts and clearer contrasts between vocalic targets [u] and [a] [73]. However, did these changes exclusively correspond to formant shift or also to their modulation? Three more results address this question.

We tested how the coefficient of variation (CV) of each formant distribution changes over the experiment; CV measures the dispersion of each formant value around its mean. F1 CVs, as F1 values, changed more in BL than CD, and, contrarily to what expected, decreased in both vocal types. However, at the end of the experiment, CD showed higher F1 CVs than BL. On the other hand, F2 values would slightly increase in CD but not in BL, potentially indicating that the learning process drove the seal to encompass more F2 values in its conditioned vocalization. The variation of the contours per se constitutes a reasonable prerequisite for vocal modulation but is not a sufficient condition. Two additional metrics directly tested for this modulation as the degree of change per time unit and its predictability through time.

Modulation Depth measured the degree of change in each formant frequency between adjacent pairs of tracked frequency values. Higher Modulation Depth means a higher change in the frequency of a formant per time unit. We found that all formants of all vocal types increased their Modulation Depth over the course of the experiment. Crucially, CD showed a higher Modulation Depth than BL for both F1 and F3. In fact, for the particular case of F3, while CD increased its Modulation Depth, BL decreased it, reflecting a divergence between the vocal types, mediated by changes in both BL and CD features. These strong differential changes in F1 and F3 suggest that our training induced a broader range of formant modulation, as opposed to haphazard production of random formant values in a broad range.

Finally, Spectral Entropy measured the predictability of formant contours to address the question: how predictable does formant modulation become over the course of the experiment? As CD vocalizations are reinforced based on cycles of opening and closing the mouth, one would expect Spectral Entropy to decrease over the course of learnt modulation. Indeed, for all 3 formants of the CD, Spectral Entropy: (1) decreased over the course of the experiment, and (2) was significantly lower for CD than for BL. In other words, training of CD triggers a decrease in Spectral Entropy; this same measure remains constant or even increases (F1) in the BL vocal type, again indicating a process of change of and separation between vocal types, for all formants.

A fifth set of analyses, partly orthogonal to the ones mentioned above, employed machine learning techniques to compare classification accuracy between vocal types from the start to the end of the experiment. Based on the modulation measures of F1 and F3, the acoustic space occupied by the two vocal types diverged significantly at the end compared to the beginning of the experiment: i.e. the algorithm correctly classified vocal types better at the end of the experiment based on F1 and F3 modulation alone.

### Overall significance and relation to previous work

To summarize, we find that training a seal to open and close its mouth while phonating: (1) is possible, (2) results in learnt vocal output where formants are modulated. Moreover, (3) the conditioned output differs from baseline vocalizations, and (4) the separation between vocal types appears to be mediated by an interconnected system of changes involving both baseline and conditioned vocalizations. These findings are not obvious for a number of reasons. First, a harbor seal can learn to open and close its mouth while phonating. Not all mammals have the necessary neural wiring to learn to control their upper vocal tract; our behavioral finding here dovetails with recent neurobiological evidence, showing that phocid seals have, among pinnipeds, some of the most apt white matter connectivity to make this happen [29]. Second, we find that this upper vocal tract motion results in a vocal output where formants are modulated. This may seem obvious from a physical and biomechanical perspective: in mammalian, including human, models of formant production, the upper vocal tract is modeled as a tube that enhances or suppresses some frequencies. Shortening or lengthening the tube results in higher and lower formants respectively. Why is this finding interesting if fully predicted by physics, at least for many terrestrial mammals? Adaptations to underwater life can lead to vocal tracts and phonatory modes that are either similar or radically different from the standard mammalian model [74, 75]; among aquatic mammals, pinnipeds have often been neglected, hence a mechanistic link between vocal mechanics and phonation similar to our own bears relevance to the broader mammalian bioacoustics debate [31]. In addition, a carnivore’s vocal tract is poorly approximated by a cylindrical tube; in a way our findings show that the standard tube model, no matter its simplicity, still describes upper vocal tract filtering in pinnipeds quite well. Third, the seal’s conditioned vocal output differs from a baseline vocalization not being subject to a targeted process of shaping, in terms of formant values but also modulation measures. In particular, we see that the two vocalization types changed over the experiment individually, but also became more different from each other. In other words, formants of CD and BL “evolved” over time both at the level of vocal type and as an interconnected system. This is particularly interesting especially given how they were trained: While the CD type was reinforced for meeting specific motor constraints, the BL type was reinforced as long as it was not the CD type. In some cases (F2 and F3 values, F1 and F2 modulation depth), the two vocal types would follow the same trend in modulation that was expected for CD. However, CD showed the steepest change and/or higher modulation at the end. We can speculate that this baseline drift would have taken place independently of the training process, and/or that the training process somehow unintendedly interfered with the vocal output of both vocal types.

From a broad perspective, here we replicate the findings of previous work finding formant modulation in a harbor seal [40]. However, from a finer grained perspective, we introduce a few modifications. In fact, (1) we tested a different individual in its adulthood, suggesting that comparable training on two harbor seals with fairly different backgrounds can lead to similar experimental outcomes. (2) The seal was tasked with producing two, as opposed to one, target vocal types, allowing for a baseline comparison with vocalization not targeted by experimental training, hence making a stronger argument for volitional control. For example, this showed that the seal not only increasingly modulated formants of CD vocalizations over the course of experiment but also decreased the modulation of BL vocalizations. In addition, (3) vocalizations were tracked longitudinally over a period of about 6 months, providing acoustic snapshots of the result of the vocal training. Moreover, (4) the resulting data allowed for extraction of the third formant, in addition to F1 and F2 also extracted in Goncharova et al., 2024; based on our results, F3 indeed explained a good portion of the variance, similarly to what happens for some human speech sounds. Finally, (5) these formants were analyzed using a suite of methods, highlighting more nuanced hypothesis-testing than in previous work; for instance, if two sounds have the same Modulation Depth, the one with higher CV will also show more formant modulation and less formant-related noise.

### Open questions

The BL vocal type, before having been placed under the control of the discriminative vocal cue, had the advantage of being a set of spontaneous vocalizations, hence encompassing a wide range of sounds freely produced by the animal. Because of this, BL sets the bar quite high for a comparison of modulation with respect to CD. However, even with these features, BL and CD ended up differing in most of the calculated modulation measures on F1 and F3 at the end of the experiment: the seal learnt to shape its vocal tract movements in time, resulting in sounds which went from a fairly “flat” vocalization to a temporally-modulated one. We observed an overall increase in modulation in most of the BL measures. Since the targeted shaping of CD may have unintentionally interfered with the reinforcement of BL, future work could explore the use of a real-time formant dynamics detector. A system of this kind - similar to that described in [35] - could integrate a Python and Parselmouth application to perform real-time acoustic analyses and provide immediate feedback on the vocalizations acoustic properties. This would offer an objective approach to reinforce specific vocal outputs. In addition, one could continue recording and monitoring BL formant features over the coming months to assess whether acoustic features would change back to the original ones, now that CD vocalizations are no longer being reinforced. Indeed, in the absence of operant conditioning shaping formant modulation, BL would probably return to the pre-existing features, as formant frequencies are, of all acoustic features, typically stable [76, 77].

Our results contained a fairly unexpected and unusual feature: the clarity and strong explanatory power of F3. First of all, F3 is in our experience more laborious to extract compared to F1 and F2. Because of this, most work in mammalian bioacoustics and speech sciences often focuses only on F1 and F2. This was the case also for the only available previous work quantifying formant modulation in the same species [40]. However, in our case, F3 had a clear shape and waseasier to track than F2: estimated sections of poorly tracked regions of the contours were less conspicuous in F3 than in F2. Despite the integration of previously adopted techniques for formant extraction with additional methodological precautions (visual inspection of formant contours, corresponding fine-tuning of formant extraction parameters, statistical elimination of outliers, simulations of different degrees of well tracked contours shuffling), we cannot rule out tracking accuracy artifacts on F2 and F3. Perhaps as a byproduct of this, we see another unexpected finding: In our data, F3 explains much of the change over the experiment and between vocal types. Conversely, perhaps expectedly, F2 is only significantly different between conditions in some of the tests. On an articulatory level, this is consistent with evidence from human speech, where vowels like [a] and [u] primarily differ along the open-close (vertical) dimension of articulation rather than the front-back (horizontal) axis [73]. In particular, in humans, F1 is correlated to the open-close axis and F2 to the front-back axis [73]. Since our training protocol targeted modulation along the open-close axis, reflected in changes to F1 modulation, the limited F2 modulation aligns with this articulatory focus and mirrors the human pattern for these particular vowel-like sounds. It is then reasonable that, with this upper vocal tract movement, F1 modulation should explain more of the data than F2. The decrease of F2 values throughout the experiment suggests that, even though secondarily, front-back concurrent articulatory adjustments might also take place [73]. Overall, however, our findings are in line with previous evidence showing a strong correlation between jaw opening and F1 in another trained harbor seal [40]. How about F3? In humans, F3 also correlates with tongue shape during phonation. While our training targeted the movement and not the formants per se, one could hypothesize that a tongue movement was introduced for one of two reasons [78]. It could be an ancillary movement, partly supporting the task of mouth opening/closing. Alternatively, a tongue movement could be a byproduct of mouth movements. Ideally, an ultrasound probe placed sagittally on the jaw during sound production could pinpoint to the mechanistic bases of the F3 modulation which we measured acoustically.

## Conclusions

With this case study, we present new evidence supporting the ability of nonhuman mammals to learn to modify formant structure via operant conditioning. While formant modulation has largely been studied in birds (e.g [79]), it is more rarely dissected in mammals. The formant modulation demonstrated in our experimental design aligns with sporadic prior findings in other mammals, such as an Asian elephant that used its trunk to modify its oral cavity [80] and gray seals that learned to adjust formants [81] to imitate human-like vowels. Although the ecological relevance of this ability is still unclear and could be connected with vocal displays in adult males [38, 82–86], operant conditioning of vocalizations provides insight into its *potential*. In other words, we could show not what an animal typically does, but what this animal is capable of doing. We emphasize that our case study, conducted on a single individual (in addition to evidence on a second individual, published in [40]), does not claim to generalize to species-wide ability, but rather falls within the realm of existence proof. Formant production requires fine-tuned vocal motor mechanical processes, learning requires specific - and rare across mammals - neurobiological processes: evidence for learned formant modulation in this species provides explicit evidence of the link between those processes and demonstrates that such a link is possible in the presented context. If supported from a wider sample of individuals and in ecologically relevant contexts, these results would indicate that upper vocal tract control, responsible for learned adjustments in formants, the primary carriers of information in human speech, is more widespread than previously surmise [25, 87]. Notably, prior research in nonhuman primates has shown limited success in demonstrating such vocal flexibility [e.g., 88], underscoring that phylogenetic proximity to humans is not necessary to have this capacity.

## Supplementary Information

Below is the link to the electronic supplementary material.

Supplementary Material 1

## Data Availability

The datasets and analysis code used in this study are available from the corresponding authors upon reasonable request.
