# Supplementary material for: Learnt formant modulation via upper vocal tract movements in a marine mammal
Source: Discov Anim. 2026 Jan 5;3(1):2. doi: 10.1007/s44338-025-00145-z (PMC12769615; doi:10.1007/s44338-025-00145-z)
Supplement: Supplementary file 1 — Supplementary Material 1 [file 44338_2025_145_MOESM1_ESM.docx]

Supplementary Information

Learnt formant modulation via upper vocal tract movements in a marine mammal

**S1** - Spectrograms (window length = 0.04 s, Dynamic range = 70 db) of one baseline (BD) and one conditioned (CD) vocalizations recorded on the same day (13/03/2025; last month of data collection). The three formant contours are displayed and correspond to the used extraction parameters in Praat (see Methods section for details). Note that displayed formant contours are prior to the removal of statistical outliers from the dataframe (see Methods and S2).


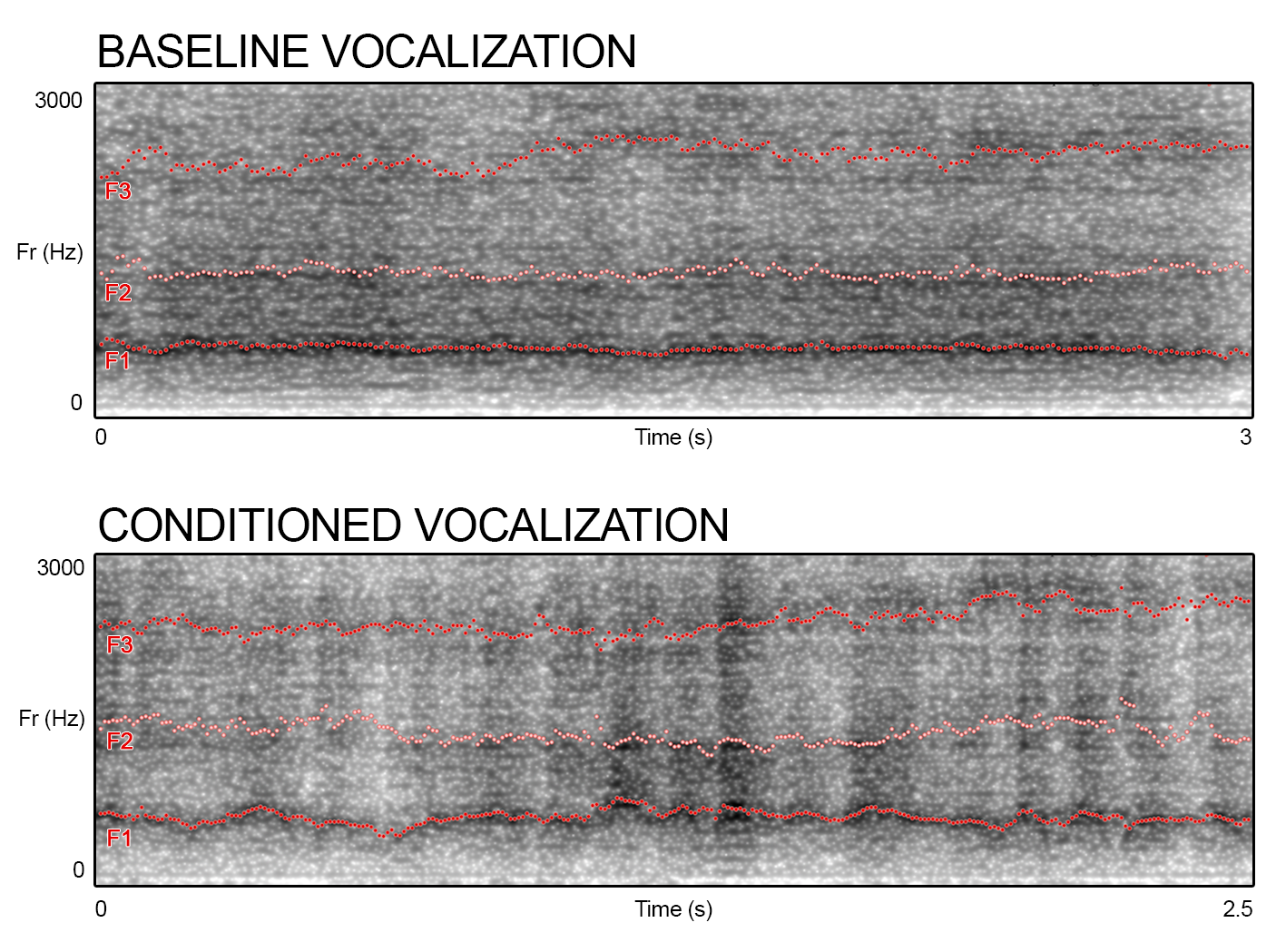


#

# **S2 -** Boxplots displaying the distribution of the acoustic variables prior (A) and after (B) the outlier removal (details in the main manuscript). In C) the summary statistics of the variables are reported.


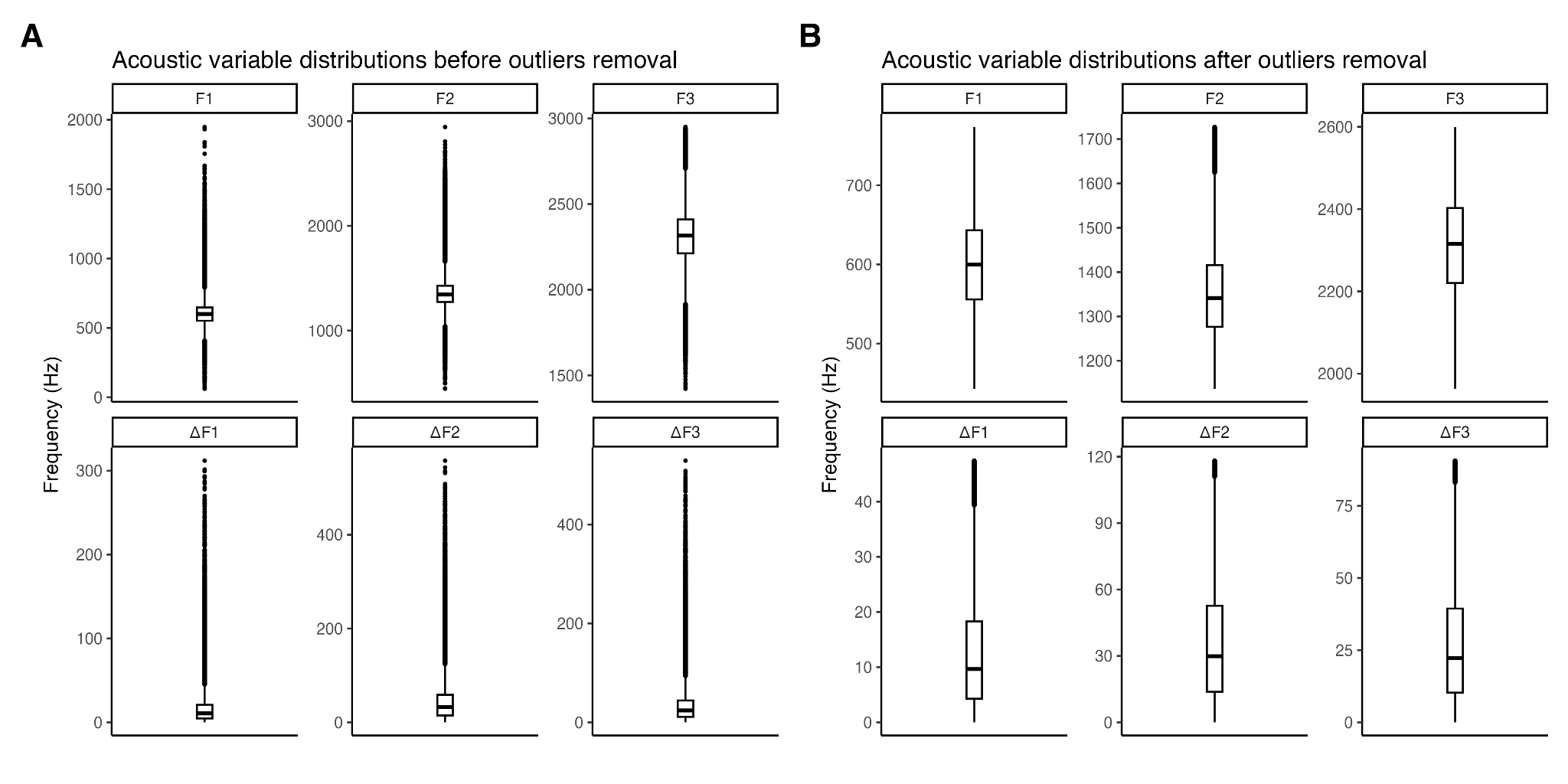


| **C** | **Before outlier removal** | | | | | | **After outlier removal** | | | | |  |  |
| --- | --- | --- | --- | --- | --- | --- | --- | --- | --- | --- | --- | --- | --- |
| **Feature** | **Min** | **1st Qu** | **Median** | **Mean** | **3rd Qu** | **Max** | **Min** | **1st Qu** | **Median** | **Mean** | **3rd Qu** | | **Max** |
| **F1** | 61.564 | 551.586 | **600.060** | 602.120 | 647.522 | 1948.305 | 443.089 | 555.862 | **599.749** | 599.228 | 643.132 | | 773.523 |
| **F2** | 444.996 | 1273.215 | **1344.569** | 1364.407 | 1428.301 | 2944.301 | 1136.906 | 1276.488 | **1341.438** | 1352.089 | 1415.680 | | 1727.253 |
| **F3** | 1422.216 | 2212.493 | **2315.867** | 2307.142 | 2410.500 | 2949.872 | 1963.518 | 2220.398 | **2315.503** | 2308.595 | 2402.648 | | 2599.553 |
| **Mod. Depth F1** | 0.000 | 4.625 | **10.629** | 15.801 | 20.911 | 312.023 | 0.000 | 4.275 | **9.657** | 12.623 | 18.300 | | 47.439 |
| **Mod. Depth F2** | 0.000 | 14.799 | **32.403** | 42.920 | 58.693 | 557.986 | 0.000 | 13.792 | **29.890** | 36.059 | 52.653 | | 118.218 |
| **Mod. Depth F3** | 0.000 | 11.052 | **24.119** | 32.634 | 44.206 | 529.272 | 0.000 | 10.292 | **22.212** | 27.116 | 39.414 | | 90.669 |

#

#

# **S3 - a)** Summary statistics and observed error of the original and shuffled contours, prior (mean, standard deviation, median, min, max, error) and after (mean_c, standard deviation_c, median_c, min_c, max_c, error_c) outliers removal (±2.5% tails, and >5% contour jumps). **b)** Dual-axis line-plots displaying the mean formant (Hz) on the left y-axis, and corresponding deviance of the observed mean from the original one on the right y-axis (%), before and after outliers removal as applied to the rest of the dataset.

# **
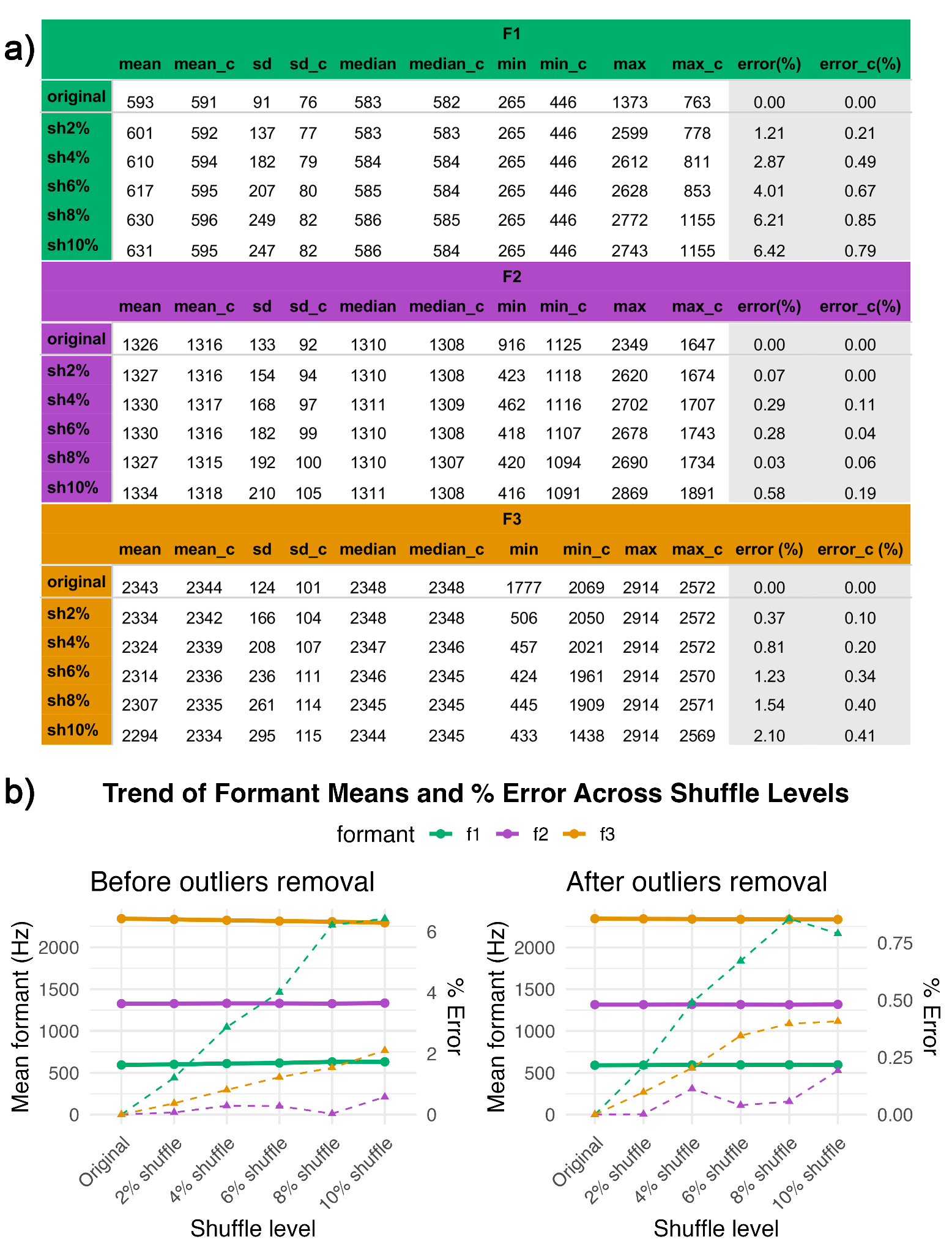
**

# **S4** - Summary table of the variables included in the statistical analyses.

| **Variable Name** | **Definition** |
| --- | --- |
| **Formant Contour Values** | Frequency values of vocal tract resonances (F1, F2, F3) at each time point of the extracted formant contours. |
| Formant contour variation (CV). | Coefficient of variation calculated for each recorded vocalization. |
| **Modulation Depth** | Calculated as the absolute value of the difference between adjacent points of a formant contour. |
| **Formant contour Spectral Entropy** | Shannon Entropy calculated on the spectrum extracted from 40 adjacent points of the formant contours. |
| **Formant** | Categorical variable referring to the specific formant. Three levels: F1, F2, F3. |
| **Vocal Type** | Categorical variable indicating the vocalization type. Two levels: *BL,* baseline vocalization; CD, conditioned vocalization. |
| **Day number** | Day of recording during the training period. Ordinal variable: days numbered sequentially from the start of training. |
| **Session Code** | Unique code assigned to each training session (2 per day). |
| **Vocalization Code** | Unique code assigned to each labelled vocalization within a training session. |

#

# **S5 -** Summaries of the three LMMs testing for the effect of time and vocal type on the formant contour values (one model for each formant). For each formant, on top, summary of the LMM testing for the effect of vocal type (BL, CD) and time on the formant values. When the interaction term was kept in the model, below each summary, slopes per vocal type are provided.

| **Term** | **Estimate** | **Std. Error** | **z value** | **Pr(>\|z\|)** |
| --- | --- | --- | --- | --- |
| 1. **SUMMARY OF THE MODEL**   **F1**  **Full = F1 contour values (transformed) ~ Vocal type * Day number + (1\|vocalization code)**    Full vs. Null (AIC_full_ = 801840.9; AIC_null_ =801947.6): Chisq = 112.735. p<0.001 | | | | |
| (Intercept) | 0.001 | 0.060 | a | a |
| Vocal type (CD)^b,c^ | -0.038 | 0.081 | -0.466 | 0.641 |
| **Day number** | **0.003** | **0.001** | **4.532** | **p<0.001** |
| **Vocal type (CD)** ^b,c^ **: Day number** | **-0.004** | **0.001** | **-4.628** | **p<0.001** |
| 1. **SLOPES PER GROUP (interactions)** | | | | |
| **Vocal Type** | **Slope** | **SE** | **95% CI** | |
| **BL** | **0.003** | **0.001** | **[0.001, 0.004]** | |
| CD | -0.001 | 0.000 | [-0.002, 0.000] | |
| 1. **SUMMARY OF THE MODEL**   **F2**  **Full = F2 contour values (transformed) ~ Vocal type + Day number + (1\|vocalization code)**    Full vs. Null (AIC_full_ = 838020.6; AIC_null_ = 838230.9): Chisq = 214.365, p<0.001 | | | | |
| (Intercept) | -0.463 | 0.037 | a | a |
| **Vocal type (CD) ^b,c^** | **0.204** | **0.031** | **6.600** | **p<0.001** |
| **Day number** | **0.004** | **0.000** | **13.530** | **p<0.001** |
| 1. **SUMMARY OF THE MODEL**   **F3**  **Full = F3contour values (transformed) ~ Vocal type * Day number + (1\|vocalization code)**    Full vs. Null (AIC_full_ = 776820.8; AIC_null_ = 776902.4): Chisq = 87.543, p<0.001 | | | | |
| (Intercept) | 0.352 | 0.063 | a | a |
| Vocal type (CD) ^b,c^ | -0.093 | 0.084 | -1.101 | 0.271 |
| **Day number** | **-0.005** | **0.001** | **-7.572** | **p<0.001** |
| **Vocal type (CD)** ^b,c^ **: Day number** | **0.002** | **0.001** | **2.010** | **0.045** |
| a Not shown as not having a meaningful interpretation.    b Estimate ± SE refers to the difference of the response between the reported level of this categorical predictor and the reference category of the same predictor.    c Day number reference = 0. Vocal type reference category: Vocal Type (BL, i.e., baseline vocalization). | | | | |
| 1. **SLOPES PER GROUP (interactions)** | | | | |
| **Vocal Type** | **Slope** | **SE** | **95% CI** | |
| **BL** | **-0.004** | **0.001** | **[-0.006, -0.003]** | |
| **CD** | **-0.003** | **0.000** | **[-0.004, -0.002]** | |

# **S6 -** Summary of the LMM testing for the effect of vocal type (BL, CD), formant (F1, F2, F3), and time on the formant contours Coefficients of Variation (CVs). On top, summary of the LMM testing for the effect of vocal type (BL, CD), formant (F1, F2, F3), and time on CVs. Below, post-hoc results (slopes per group and pairwise comparison).

| **a.** **SUMMARY OF THE MODEL****Full = Coefficient of Variation (transformed) ~ Vocal Type * Formant * Day Number + (1\|session code)**Full vs. Null (AIC_full_ = 6286.572; AIC_null_ = 9216.843): Chisq = 2952.271, p<0.001 | | | | | |
| --- | --- | --- | --- | --- | --- |
| Term | | Estimate | Std. Error | z value | Pr(>\|z\|) |
| (Intercept) | | 1.017 | 0.069 | a | a |
| Vocal Type (CD) ^b,c^ | | 0.294 | 0.084 | 3.487 | < 0.001 |
| Formant (F2) ^b,c^ | | -1.084 | 0.088 | -12.334 | < 0.001 |
| Formant (F3) ^b,c^ | | -2.010 | 0.088 | -22.855 | < 0.001 |
| Day number | | -0.007 | 0.001 | -10.611 | < 0.001 |
| Vocal Type (CD) ^b,c^ : Formant (F2) ^b,c^ | | -0.017 | 0.118 | -0.142 | 0.887 |
| Vocal Type (CD) ^b,c^ : Formant (F3) ^b,c^ | | -0.242 | 0.118 | -2.050 | 0.040 |
| Vocal Type (CD) ^b,c^ : Day number | | 0.005 | 0.001 | 6.320 | < 0.001 |
| Formant (F2) ^b,c^ : Day number | | 0.007 | 0.001 | 7.845 | < 0.001 |
| Formant (F3) ^b,c^ : Day number | | 0.007 | 0.001 | 7.985 | < 0.001 |
| Vocal Type (CD) ^b,c^ : Formant (F2) ^b,c^ : Day number | | -0.003 | 0.0011 | -2.645 | 0.008 |
| Vocal Type (CD) ^b,c^ : Formant (F3) ^b,c^ : Day number | | -0.003 | 0.001 | -3.137 | 0.002 |
| ^a^ Not shown as not having a meaningful interpretation.^b^ Estimate ± SE refer to the difference of the response between the reportedlevel of this categorical predictor and the reference category of the samepredictor.^c^ Day number reference = 0. Vocal type reference category: Vocal Type (BL, i.e., baseline vocalization).Formant reference category: F1. | | | | | |
| **b.** **SLOPES PER GROUP** | | | | | |
| Formant | Vocal type | Slope | SE | 95% CI | |
| F1 | **BL** | **-0.0071** | **0.0007** | **[-0.0084, -0.0058]** | |
|  | **CD** | **-0.0021** | **0.0006** | **[-0.0033, -0.0009]** | |
| F2 | BL | -0.0005 | 0.0007 | [-0.0018, 0.0008] | |
|  | **CD** | **0.0016** | **0.0006** | **[0.0004, 0.0027]** | |
| F3 | BL | -0.0004 | 0.0007 | [-0.0017, 0.0009] | |
|  | CD | 0.0011 | 0.0006 | [0.0000, 0.0023] | |
| **c.** **POST-HOC COMPARISONS** | | | | | |
| Formant | Contrast | Estimate | SE | t.ratio | p.value |
| **F1** | **BL - CD** | **-0.0050** | **0.0008** | **-6.320** | **<.0001** |
| F2 | BL - CD | -0.0021 | 0.0008 | -2.619 | 0.0089 |
| **F3** | BL - CD | -0.0015 | 0.0008 | -1.931 | 0.0535 |

# **S7 -** Summary of the LMM testing for the effect of vocal type (BL, CD), formant (F1, F2, F3), and time on the formant contour Modulation Depth. On top, summary of the LMM testing for the effect of vocal type (BL, CD), formant (F1, F2, F3), and time on the formant Modulation Depth. Below, post-hoc results (slopes per group and pairwise comparison).

| 1. **SUMMARY OF THE MODEL**   **Full = Modulation Depth (transformed) ~ Vocal Type * Formant * Day Number + (1\|vocalization code)**  Full vs. Null (AIC_full_ = 2462030; AIC_null_ = 2641797): Chisq = 179789.1, p<0.001 | | | | | | | | |
| --- | --- | --- | --- | --- | --- | --- | --- | --- |
| Term | | | Estimate | | Std. Error | z value | | Pr(>\|z\|) |
| (Intercept) | | | -0.629 | | 0.009 | a | | a |
| Vocal Type (CD) ^b,c^ | | | -0.005 | | 0.012 | -0.38 | | 0.701 |
| Formant (F2) ^b,c^ | | | 0.968 | | 0.006 | 152.67 | | < 0.001 |
| Formant (F3) ^b,c^ | | | 0.748 | | 0.006 | 117.31 | | < 0.001 |
| Day number | | | 0.001 | | 0.000 | 2.96 | | 0.003 |
| Vocal Type (CD) ^b,c^ : Formant (F2) ^b,c^ | | | 0.101 | | 0.009 | 11.52 | | < 0.001 |
| Vocal Type (CD) ^b,c^ : Formant (F3) ^b,c^ | | | -0.005 | | 0.009 | -0.61 | | 0.540 |
| Vocal Type (CD) ^b,c^ : Day number | | | 0.002 | | 0.000 | 14.68 | | < 0.001 |
| Formant (F2) ^b,c^ : Day number | | | 0.001 | | 0.000 | 8.69 | | < 0.001 |
| Formant (F3) ^b,c^ : Day number | | | -0.001 | | 0.000 | -8.88 | | < 0.001 |
| Vocal Type (CD) ^b,c^ : Formant (F2) ^b,c^ : Day number | | | -0.002 | | 0.000 | -20.31 | | < 0.001 |
| Vocal Type (CD) ^b,c^ : Formant (F3) ^b,c^ : Day number | | | -0.001 | | 0.000 | -5.87 | | < 0.001 |
| ^a^ Not shown as not having a meaningful interpretation.  ^b^ Estimate ± SE refer to the difference of the response between the reported  level of this categorical predictor and the reference category of the same  predictor.  ^c^ Day number reference = 0. Vocal type reference category: Vocal Type (BL, i.e., baseline vocalization).  Formant reference category: F1. | | | | | | | | |
| 1. **SLOPES PER GROUP** | | | | | | | | |
| Formant | Vocal type | Slope | | SE | | 95% CI | | |
| F1 | **BL** | **0.0003** | | **0.0001** | | **[0.0010, 0.0040]** | | |
|  | **CD** | **0.0020** | | **0.0001** | | **[0.0018, 0.0021]** | | |
| F2 | **BL** | **0.0008** | | **0.0001** | | **[0.0006, 0.0010]** | | |
|  | **CD** | **0.0007** | | **0.0001** | | **[0.0006, 0.0009]** | | |
| F3 | **BL** | **-0.0003** | | **0.0001** | | **[-0.0005, -0.0001]** | | |
|  | **CD** | **0.0009** | | **0.0001** | | **[0.0008, 0.0011]** | | |
| 1. **POST-HOC COMPARISONS** | | | | | | | | |
| Formant | Contrast | Estimate | | SE | | t.ratio | p.value | |
| **F1** | **BL - CD** | **-0.0017** | | **0.0001** | | **-14.684** | **<.0001** | |
| F2 | BL - CD | 0.0001 | | 0.0001 | | 0**.**707 | 0.4798 | |
| **F3** | **BL - CD** | **-0.0012** | | **0.0001** | | **-10.198** | **<.0001** | |

# **S8 -** Summary of the LMM testing for the effect of vocal type (BL, CD), formant (F1, F2, F3), and time on the formant Spectral Entropy. On top, summary of the LMM testing for the effect of vocal type (BL, CD), formant (F1, F2, F3), and time on the spectral entropy of the formant contours. Below, post-hoc results (slopes per group and pairwise comparison).

| 1. **SUMMARY OF THE MODEL**   **Full = Spectral Entropy (transformed) ~ Vocal type * Formant * Day number + (1\|session code)**  Full vs. Null (AIC_full_ = 43950.77; AIC_null_ = 46264.36): Chisq = 2335.593, p<0.001 | | | | | |
| --- | --- | --- | --- | --- | --- |
| **Term** | **Estimate** | **Std. Error** | **z value** | **Pr(>\|z\|)** | |
| (Intercept) | -0.418 | 0.045 | a | a | |
| Formant (F2) ^b,c^ | 0.914 | 0.043 | 21.087 | < 0.001 | |
| Formant (F3) ^b,c^ | 0.399 | 0.043 | 9.290 | < 0.001 | |
| Vocal type (CD) ^b,c^ | -0.166 | 0.044 | -3.769 | < 0.001 | |
| Day number | 0.003 | 0.000 | 6.569 | < 0.001 | |
| Formant (F2)^b,c^ : Vocal type (CD)^b,c^ | -0.002 | 0.067 | -0.026 | 0.979 | |
| Formant (F3)^b,c^ : Vocal type (CD)^b,c^ | 0.010 | 0.063 | 0.153 | 0.878 | |
| Formant (F2)^b,c^ : Day number | -0.002 | 0.000 | -5.077 | < 0.001 | |
| Formant (F3)^b,c^ : Day number | -0.003 | 0.000 | -7.719 | < 0.001 | |
| Vocal type (CD)^b,c^ : Day number | -0.004 | 0.001 | -6.913 | < 0.001 | |
| Formant (F2)^b,c^ : Vocal type (CD)^b,c^:Day number | 0.002 | 0.001 | 1.939 | 0.053 | |
| Formant (F3)^b,c^ : Vocal type (CD) ^b,c^ : Day number | 0.002 | 0.001 | 2.009 | 0.044 | |
| ^a^ Not shown as not having a meaningful interpretation.    ^b^ Estimate ± SE refer to the difference of the response between the reported level of this categorical predictor and the reference category of the same predictor.    ^c^ Vocal type reference category: BL, i.e., baseline vocalization. Formant reference category: F1. Day number reference = 0. | | | | | |
| 1. **SLOPES PER GROUP** | | | | | |
| Formant | Vocal type | Slope | SE | 95% CI | |
| F1 | **BL** | **0.003** | **0.000** | **[0.002, 0.004]** | |
|  | **CD** | **-0.001** | **0.001** | **[-0.003, -0.001]** | |
| F2 | BL | 0.001 | 0.000 | [-0.000, 0.002] | |
|  | **CD** | **-0.002** | **0.001** | **[-0.003, -0.001]** | |
| F3 | BL | 0.000 | 0.000 | [-0.001, 0.008] | |
|  | **CD** | **-0.003** | **0.001** | **[-0.005, -0.002]** | |
| 1. **POST-HOC COMPARISONS** | | | | | |
| Formant | Contrast | Estimate | SE | t.ratio | p.value |
| **F1** | **BL - CD** | **0.004** | **0,001** | **6.913** | **< 0.001** |
| **F2** | **BL - CD** | **0.002** | **0,001** | **4.228** | **< 0.001** |
| **F3** | **BL - CD** | **0.002** | **0,000** | **4.523** | **< 0.001** |

# **S9 -** Summary table of Random Forest Classification for the start vs. end of the experiment.

| **Parameter** | **START (Q1)** | | | | **END (Q4)** | | |
| --- | --- | --- | --- | --- | --- | --- | --- |
| **Random Forest Classification**  **randomForest(Vocal Type ~ [V1, V2], ntree=800)**  **where V1 and V2 are the UMAP extracted dimensions** | | | | | | | |
| **Training** | | | | | | | |
| **OOB*** | 0.49 | | | 0.08 | | | |
| **Class error (BL)*** | 0.54 | | | 0.08 | | | |
| **Class error (CD)*** | 0.45 | | | 0.08 | | | |
| **Confusion matrices*** |  | BL | CD |  | | BL | CD |
|  | BL | 24 | 28 | BL | | 59 | 5 |
|  | CD | 27 | 33 | CD | | 3 | 33 |
| **Testing** | | | | | | | |
| **Confusion matrices*** |  | BL | CD |  | | BL | CD |
|  | BL | 8 | 8 | BL | | 21 | 3 |
|  | CD | 11 | 21 | CD | | 4 | 16 |
| **AUC**  Delong test result:  D = -3.145,  df = 41.441, p-value = 0.003 | 0.64 | | | 0.90 | | | |
| **Balanced Accuracy** | 0.58 | | | 0.84 | | | |
| **Sensitivity/Recall** | 0.66 | | | 0.80 | | | |
| **Specificity** | 0.50 | | | 0.87 | | | |
| (*) output that depends on prevalence, on the class distribution and/or size of the dataset: not reliable for comparison across different sample sets; to be used comparatively but not as absolute indicators. | | | | | | | |
